# Enhanced Characterization of Lysine-Linked Antibody Drug Conjugates Enabled by Middle-Down Mass Spectrometry and Higher-Energy Collisional Dissociation-Triggered Electron-Transfer/Higher-Energy Collisional Dissociation and Ultraviolet Photodissociation

**DOI:** 10.3390/antib13020030

**Published:** 2024-04-17

**Authors:** Eleanor Watts, Aarti Bashyal, Sean D. Dunham, Christopher M. Crittenden, Jennifer S. Brodbelt

**Affiliations:** 1 Department of Chemistry, University of Texas at Austin, Austin, TX 78712, USA; eleanorwatts@utexas.edu (E.W.); abashyal@utexas.edu (A.B.);; 2 Small Molecule Pharmaceutical Sciences, Genentech Inc., South San Francisco, CA 94080, USA

**Keywords:** antibody drug conjugate, trastuzumab-emtansine, tandem mass spectrometry, payload, biotherapeutic

## Abstract

As the development of new biotherapeutics advances, increasingly sophisticated tandem mass spectrometry methods are needed to characterize the most complex molecules, including antibody drug conjugates (ADCs). Lysine-linked ADCs, such as trastuzumab-emtansine (T-DM1), are among the most heterogeneous biotherapeutics. Here, we implement a workflow that combines limited proteolysis with HCD-triggered EThcD and UVPD mass spectrometry for the characterization of the resulting middle-down large-sized peptides of T-DM1. Fifty-three payload-containing peptides were identified, ranging in mass from 1.8 to 16.9 kDa, and leading to the unambiguous identification of 46 out of 92 possible conjugation sites. In addition, seven peptides were identified containing multiple payloads. The characterization of these types of heterogeneous peptides represents an important step in unraveling the combinatorial nature of lysine-conjugated ADCs.

## 1. Introduction

Antibody–drug conjugates (ADCs) represent a promising class of therapeutic treatments. The combination of a selective antibody and cytotoxic payload in the form of an ADC has been hailed as a “magic bullet” given their promise to dramatically improve treatments [1,2]. While a variety of different modalities, including site-specific ADCs, are under development and in clinical trials, all ADCs that have received FDA approval fall into the categories of either cysteine- or lysine-linked ADCs [3]. The use of either reduced cysteines or solvent-accessible lysine residues as conjugation sites adds heterogeneity to the antibodies, increasing the challenges of characterization. Recent advances in mass spectrometry techniques have resulted in enhanced structural characterization of ADCs as well as improved differentiation of positional isomers with middle-down techniques [4,5]. However, the translation of these methods to lysine-linked ADCs has been limited.

The complexity of an ADC and the difficulty associated with its characterization arises largely from the modality of the payload-to-antibody linkage. The least complex cases are site-specific or next-generation ADCs, which utilize modified amino acid residues, typically on the fragment crystallizable (Fc) domain of the heavy chain (HC), to link the payload to a small number (two or four) of specific amino acids [6]. Cysteine-linked ADCs typically contain an average of four payloads attached to interchain disulfide bonds, resulting in eight possible conjugation sites per ADC [6]. Finally, lysine-linked ADCs are derived from linker conjugation to random lysine residues along the entire amino acid sequence of the antibody [6]. The large number of lysine residues on antibodies, typically around 90 in total, increases the challenge of characterizing lysine-linked ADCs.

Despite the challenges, significant strides have been made to improve the characterization of ADCs. Until recently, drug-to-antibody ratios (DARs) were typically measured through liquid chromatography coupled to UV–visible spectroscopy, and bottom-up proteomic methods were the only feasible means to identify the locations of payload binding sites [7,8]. Enhanced native mass spectrometry, chromatographic methods, and the increased availability of high-resolution mass spectrometry instrumentation have facilitated more advanced characterization of intact ADCs through intact mass spectrometry, making it the new gold standard for DAR assessment [9,10,11,12,13,14,15,16,17,18,19]. Moreover, recent developments in ion-mobility and hydrogen–deuterium exchange mass spectrometry have further elevated the capabilities of mass spectrometry for structural characterization of ADCs [20,21,22,23,24,25,26,27]. Finally, the growing prevalence of subunit-based middle-down strategies has eliminated the notion that drug conjugation site identification can only be achieved with bottom-up proteomics [4,5,28,29]. Despite the prolific achievements described thus far, very few studies have translated the successes of intact and structural characterization or middle-down mass spectrometry to lysine-linked ADCs. Moreover, with the ongoing evolution of ADCs that aim to enhance conjugation site-specificity [1,2,3], the exploration of alternative analytical methods remains timely.

Given the complexity associated with lysine-linked ADCs, bottom-up mass spectrometry remains the primary method to identify payload locations. Varying numbers of payloads have been identified for lysine-linked ADCs [30]. Most studies report an average of 40 out of 92 possible sites identified, while one reported 82 conjugation sites [31,32,33,34]. These studies typically rely on digestion with trypsin, resulting in peptides containing only one possible lysine conjugation site per peptide, which greatly simplifies the localization of the payloads but eliminates all chances of identifying combinatorial modifications. Most bottom-up ADC studies use collisionally activated dissociation (CAD) for the characterization of the peptides which, in addition to generating sequence ions, may result in fragmentation of the labile payload or its cleavage from the ADC [31,32,33,34]. The generation of highly abundant payload-related fragment ions has been reported for the CAD of ADCs containing emtansine (DM1), the payload commonly used in lysine-linked ADCs [31,32,33,34]. The presence of these fragment ions has been exploited to unambiguously detect the presence of payload-containing peptides [31,32,33,34]. This feature becomes a significant attribute in the development of CAD-based methods that aim to screen digests for the presence of payload-containing peptides, as utilized in the present study.

While bottom-up proteomics methods have proven successful for identifying payload locations, they are frequently unable to capture the full heterogeneity of ADCs nor unravel the context of multiple co-existing payload locations. Employing a middle-down approach in which larger peptides are generated and analyzed is a promising option for the improved characterization of complex biomolecules [35,36,37,38,39,40,41,42]. Several studies have now reported the characterization of site-specific, cysteine-linked, and even lysine-linked ADCs using middle-down strategies enabled by IdeS or IdeZ proteases which cleave ADCs into large subunits [4,5,29]. Despite the achievements of these studies, the characterization of the lysine-linked ADCs at the subunit level remains hampered by the increased complexity compared to the cysteine-linked counterparts [5]. The limitations of subunit-level characterization can be subverted by employing alternative proteases or conditions to modulate the peptide sizes, generating ones larger than tryptic peptides but smaller than intact subunits. Limited proteolysis has resulted in improved characterization of proteins, including monoclonal antibodies [36,42,43]. This approach could augment the characterization of lysine-linked ADCs by allowing for the generation of longer peptides representative of proteoforms containing multiple drug linkages and is adopted in the present study.

Mass spectrometric characterization of ADCs relies on proficient tandem mass spectrometry (MS/MS) methods to generate informative fragmentation patterns to map the antibody sequences and localize the attached drugs [7,8]. Collision-based dissociation methods are the most well established for the identification of peptide sequences. Recently, alternative MS/MS methods, including electron-transfer/higher-energy collision dissociation (EThcD) and ultraviolet photodissociation (UVPD) have gained popularity. EThcD is a hybrid method combining electron-transfer dissociation (ETD) and CAD to enhance the conversion of charge-reduced peptides into diagnostic *b*/*y* and *c*/*z* fragment ions along with retention of labile modifications [44]. UVPD is a higher-energy activation method that causes extensive fragmentation of peptides and proteins and also allows for the retention of labile PTMs [45]. The application of these MS/MS methods to lysine-linked ADCs is highlighted in the present investigation.

In this study, we focus on advancing the characterization of lysine-linked ADCs, as exemplified by trastuzumab-emtansine (T-DM1, brand name Kadcyla), via a middle-down strategy that utilizes limited proteolysis with lysyl-endopeptidase (Lys-C). To increase the throughput of the liquid chromatography–MS/MS workflow and the confidence in the identification of payload-containing peptides, a higher-energy collisional dissociation (HCD)-triggered method is employed using payload-related reporter ions, which have already been identified for DM1 to trigger a second high-resolution MS^2^ event utilizing either UVPD or EThcD. The performance of the two auxiliary MS/MS methods is compared in the context of localizing multiple payloads.

## 2. Materials and Methods

### 2.1. Sample Preparation

All reagents were purchased from Thermo-Fisher Scientific (San Jose, CA, USA) unless otherwise noted. Lyophilized lysine-linked antibody–drug conjugate (T-DM1) samples (20 mg/mL) were provided by Genentech (San Francisco, CA, USA). For middle-down analysis, the T-DM1 samples were diluted or reconstituted in water to a concentration of approximately 2.5 mg/mL and then desalted with Micro Bio-Spin 6 columns (Bio-Rad, Hercules, CA, USA) prior to Lys-C digestion. After cleanup, samples were diluted to 1 mg/mL in 50 mM sodium phosphate pH 7.5 (New England Biolabs, Ipswich, MA, USA). Denaturation prior to digestion was completed using 2 M urea and 10 mM tris(2-carboxyethyl)phosphine (TCEP). Limited proteolysis was achieved by adding Lys-C (Santa Cruz Biotechnology, Dallas, TX, USA) in a 1:75 enzyme-to-protein ratio and digesting for 2 h at 37 °C. Complete reduction was ensured after digestion by the addition of additional 50 mM TECP and 15 min of shaking at room temperature. Samples were diluted in water and acidified with 0.1% formic acid prior to analysis by LC-MS/MS.

For bottom-up analysis, 50 µg of different lots of ADCs was reduced with 5 mM dithiothreitol in the presence of 2 M urea at 55 °C for 40 min. Samples were alkylated with 15 mM iodoacetamide for 30 min at room temperature in the dark and were digested overnight with trypsin (1% by weight) at 37 °C in a final volume of 60 µL in 0.1 M Tris at pH 7.5. Peptides were desalted using C18 spin columns and reconstituted in 200 μL water.

### 2.2. Liquid Chromatography-Mass Spectrometry

Liquid chromatography-mass spectrometry was performed using a Dionex Ultimate nano liquid chromatography system (San Jose, CA, USA) coupled to a Thermo Scientific Instruments Orbitrap Fusion Lumos mass spectrometer (San Jose, CA, USA) equipped with 193 nm UVPD as previously described [46]. For the middle-down analysis of the Lys-C digests, chromatographic separation was achieved with trap and elute using columns house-packed with Agilent (Santa Clara, CA, USA) polymeric reversed-phase (PLRP-S) bulk media. Trap (100 µm ID/360 µm OD) and analytical columns (75 µm ID/360 µm OD) were packed with 5 μm bulk PLRP media (1000 Å pore size) from Agilent to a length of 3 and 20 cm, respectively. Samples (0.5 µg) were injected under starting conditions of 2% acetonitrile and 0.1% formic acid in water at 5 μL/min. After 5 min of loading, a valve switch placed the trap column in line with the analytical column. Analytical mobile phases comprising water with 0.1% formic acid (A) and acetonitrile with 0.1% formic acid (B) were applied at a rapid initial gradient of 2 to 20% B over 2 min followed by a slower gradient up to 40% B over 33 min at a flow rate of 300 nL/min.

Upon chromatographic separation of the Lys-C digests, each eluate was introduced to the mass spectrometer by electrospray ionization with an applied voltage of 2 kV. MS1 spectra were collected with a resolution of 60,000 at *m*/*z* 200, an AGC target of 4 × 10^5^, and a 50 ms maximum ion injection time. Data-dependent properties were set to allow for 10 scans with exclusion after two repeats within 7 s and a 30 s exclusion duration. All runs included a preliminary HCD MS^2^ event with 30% normalized collisional energy, a resolution of 30,000 at *m*/*z* 200, an AGC target of 5 × 10^5^, and a 54 ms maximum ion injection time. Targeted inclusion triggered a second MS^2^ event on the same precursor if a fragment ion of *m*/*z* 547.22 was detected in the HCD scan. The secondary MS^2^ event utilized either 193 nm UVPD with two 2 mJ laser pulses (applied during a 4 ms activation period) or EThcD with calibrated-charge-dependent activation period ranging from 3 to 150 ms and 15% normalized collisional energy supplemental activation. Secondary MS^2^ scans utilized a resolution of 240,000 at *m*/*z* 200, an AGC target of 5 × 10^5^, and a 502 ms maximum ion injection time. Five technical replicates were collected for both UVPD and EThcD.

For analysis of the tryptic digests using a conventional bottom-up approach, 500 ng of peptides were injected for LC-MS/MS analysis. Peptides were pre-concentrated in a 3 cm trap column (100 µm ID/360 µm OD) for 5 min and then separated using a 15 cm C18 analytical column (75 µm ID/360 µm OD). Mobile phase A was 0.1% formic acid in water and mobile phase B was 0.1% formic acid in acetonitrile. Peptides were eluted using a gradient of 5–35% B for 32 min for HCD runs. The gradient length was increased for the HCD-triggered approach (EThcD and UVPD) to 62 min to allow for more time between eluting peaks and to improve the identification of low-abundance peptides. For the HCD-triggered approach, detection of a diagnostic fragment ion of *m*/*z* 547.22 (generated by fragmentation of the payload) was used to trigger EThcD or UVPD events. HCD was performed at a resolution of 30,000 (at *m*/*z* 200) and the triggered EThcD or UVPD spectra were collected at a resolution of 60,000 (at *m*/*z* 200).

### 2.3. Data Analysis

For middle-down results obtained for the Lys-C digests, data analysis was performed with ProSight PD 4.2 within Proteome Discover 3.0 (Thermo Fisher Scientific, San Jose, CA, USA). To implement middle-down data processing, a custom middle-down database was generated using Protein Digestion Simulator (Pacific Northwest National Labs, Richland, WA, USA) to create a list of peptides produced from any number of missed cleavages at lysine. This list was input into Proteome Discoverer as a FASTA file. The addition of a 956.364 Da payload was included as a custom variable modification for each lysine residue and for the N-terminus of the heavy and light chains. Spectra were processed with ProSight PD High/High cRAWler and matched with ProSight PD 4.2 Annotated Proteoform search. In the cRAWler module, a fit factor of 0.80, a remainder of 0.25, and a S/N threshold of 3 were used for deconvolution of fragmentation spectra with the Xtract algorithm. For the annotated proteoforms, search precursor mass tolerance was set to 2.2 Da and fragment mass tolerance was set to 10 ppm. The “UVPD 9” setting was used to allow for inclusion of *a*, *a +* 1, *b*, *c*, *x*, *x +* 1, *y*, *y* − 1, and *z* ions in UVPD search. Payload-containing fragment ions were only considered if they contained the entire payload because very few fragment ions containing partial modifications were identified. Matches were filtered to only include those identified with “Medium Confidence” or higher. Payload-containing hits were only considered if they were matched in secondary MS^2^ scans (i.e., EThcD or UVPD) in at least three out of the five replicates and matches were individually validated. Details of the manual validation process are explained in the discussion. All data files are available in massive database (massive.ucsd.edu, 22 November 2023) with accession number MSV000092585. For reviewer access use username: MSV000092585_reviewer and password: ECWNov22.

For bottom-up results obtained for the tryptic digests, data was analyzed using Byonic using the following search parameters: up to three missed cleavages, 10 ppm precursor tolerance, and 20 ppm fragment tolerance. Oxidation of methionine, deamidation of asparagine and glutamine, amidation of aspartate and glutamate, and a mass shift of 956.36 Da (maximum 2 per peptide) to lysines were searched as variable modifications. Carbamidomethylation of cysteine was used as a static modification.

## 3. Results

### 3.1. Development of a Middle-Down Method

While specific enzymatic cleavage at the hinge region of an antibody, such as that performed using IdeS or IdeZ proteases, is a popular approach for middle-down characterization; it has limitations in localizing payloads to specific lysine residues owing to the large sizes (25–100 kDa) of the resulting subunits (i.e., Fc, Fc/2, F(ab′)_2_, Fd′) and the substantial number of lysines per subunit (13 to 52). The generation of middle-down peptides in the 3–10 kDa range has shown promise for antibodies, histones, and other proteins containing complex post-translational modifications [36,41,43]. In these prior studies, limited proteolysis was achieved using Lys-C with a high antibody-to-protease ratio and shorter digestion time than would be used for conventional bottom-up proteolysis. Because this method was successful for the generation of large peptides for the characterization of antibodies in the past [36], this strategy was adopted for analysis of T-DM1 and proved reproducible for the production of the desired target size of peptides (3–10 kDa).

While CAD (including beam-type HCD implemented on a linear ion trap and Orbitrap mass spectrometers) has been highly successful for the characterization of small peptides, such as those generated by tryptic digestion, alternative higher-energy ion activation methods like EThcD and UVPD are often better suited for the characterization of larger peptides. However, the longer signal averaging required to adequately resolve the denser fragmentation patterns generated by EThcD and UVPD relative to those by CAD reduces throughput. Methods that capitalize on the high throughput of CAD and the enhanced peptide characterization of ETD, EThcD, or UVPD have been developed and are generally known as “triggered” methods, as demonstrated previously for the analysis of phosphopeptides and glycopeptides [47,48]. In essence, the slower ETD, EThcD, or UVPD scans are only acquired if an initial CAD scan generates a reporter ion characteristic of a particular type or class of peptide. Then, a second MS/MS scan (UVPD or EThcD) is undertaken on the same precursor ion pre-identified by the first CAD or HCD scan to enable more detailed structural characterization. By only collecting a higher resolution EThcD or UVPD scan if payload-specific fragment ions are detected by CAD or HCD, the number of high-resolution scans is reduced, improving the duty cycle and maximizing the time spent analyzing the peptides of interest. Collisional activation of peptides containing the DM1 payload of T-DM1 has previously been reported to generate unique fragment ions which have also been exploited to aid in data processing [31,32,33,34]. The presumed structures of these fragment ions, with *m*/*z* values of 453.19, 485.22, and 547.22, are displayed in Scheme S1. These same ions were adopted as potential reporter ions for the triggered MS/MS method implemented in the present study.

**Figure 1:**
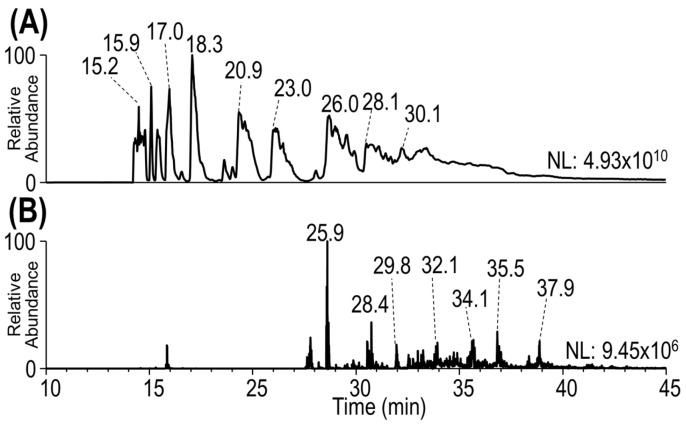
(**A**) Total ion MS^1^ chromatogram and (**B**) HCD MS^2^ extracted ion chromatogram for reporter ions of *m*/*z* 547.22, 485.22, and 453.19 from a limited proteolysis Lys-C digestion of T-DM1.

**Figure 2:**
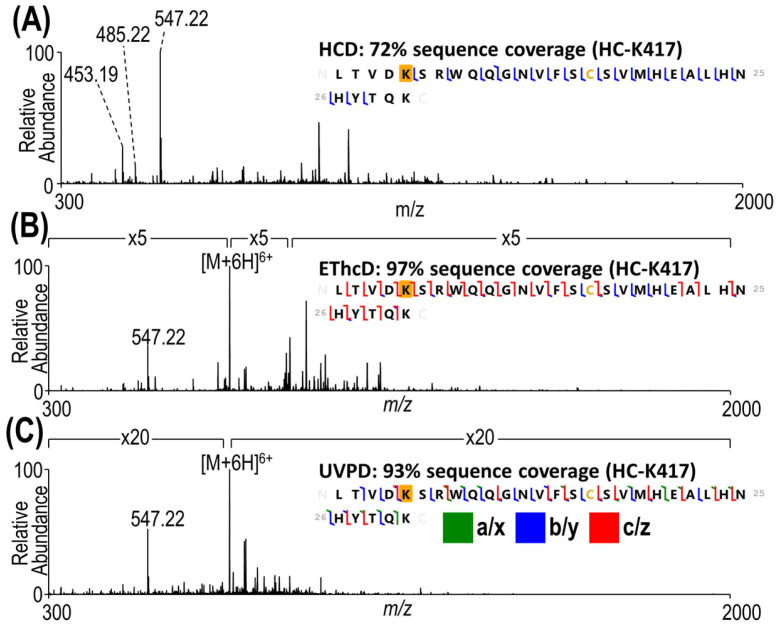
MS/MS spectra of a 4.5 kDa peptide (6+) using (**A**) HCD (30% NCE), (**B**) EThcD (charge-calibrated activation and 15% NCE supplemental activation), and (**C**) 193 nm (UVPD 2 pulses, 2 mJ per pulse). Sequence coverage maps along with sequence coverages are included for each spectrum. Lysine K5 of the peptide sequence shown here corresponds to K417 of the heavy chain of the antibody. The modified lysines are shaded in gold.

**Figure 3:**
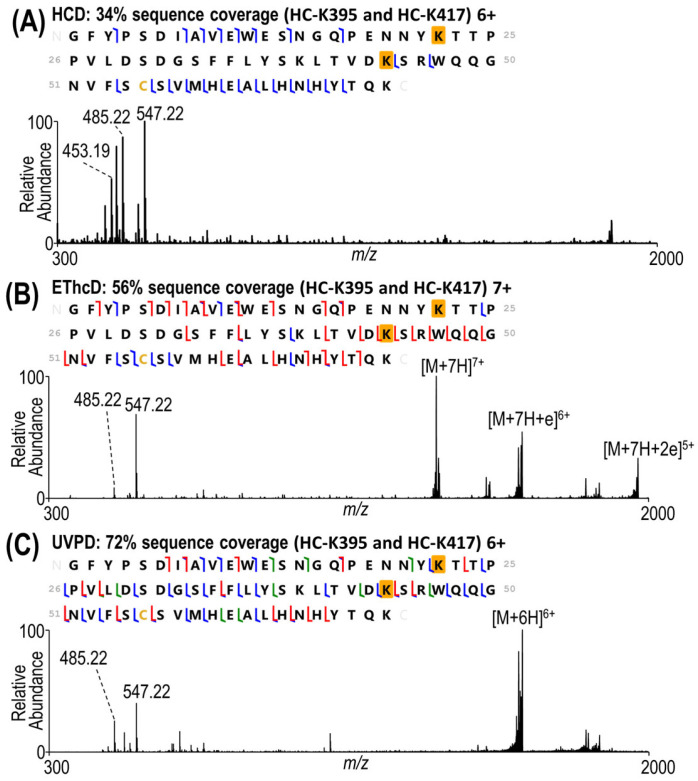
MS/MS spectra of an 9.8 kDa peptide by (**A**) HCD (30% NCE), (**B**) EThcD (charge-calibrated activation and 15% NCE supplemental activation), and (**C**) 193 nm (UVPD 2 pulses, 2 mJ per pulse). The 6+ charge state is displayed for HCD and UVPD and the 7+ charge state for EThcD in order to achieve the best characterization for each method. Sequence coverage maps along with sequence coverages are included for each spectrum. Residues K22 and K44 of the peptide sequence shown here correspond to K395 and K417 of the heavy chain of the antibody. The modified lysines are shaded in gold.

The chromatogram in Figure 1 illustrates the separation of the peptides generated by limited Lys-C proteolysis of T-DM1, along with the extracted ion chromatograms corresponding to the contribution of the payload-specific fragment ions. The total ion chromatogram in Figure 1A includes all peptides, regardless of whether they are key payload-containing peptides or not. Figure 1B displays only those peptides that produced DM1-payload-specific fragments upon HCD of the eluting peptides. Closer examination of the individual HCD mass spectra revealed that the fragment ion of *m*/*z* 547.22 was consistently the highest abundance and most prevalent of the three reporter ions, as exemplified in Figure 2A and Figure 3A. Thus, for the HCD-triggered UVPD and EThcD methods in our strategy, the *m*/*z* 547.22 reporter ion was utilized.

As expected, HCD resulted in adequate characterization of smaller peptides without multiple payload sites, as shown in the example in Figure 2A. Interestingly, even for this relatively small 4.5 kDa peptide containing 30 residues, the level of characterization by HCD is limited in that no payload-containing fragment ions are generated, indicating that the DM1 payload is labile and readily cleaved by HCD. In contrast, EThcD and UVPD (Figure 2B,C) generated many payload-containing products in addition to providing more extensive sequence coverage.

For the larger 9.8 kDa peptide containing two payloads shown in Figure 3, the enhanced fragmentation offered by EThcD and UVPD is even more beneficial. In this example, the HCD spectrum (Figure 3A) displays the abundant payload reporter ions in the low *m*/*z* range, but no fragment ions originated from backbone cleavages between any of the three lysine residues. The EThcD spectrum in Figure 3B offers higher sequence coverage (56%), including eight one-payload- and six two-payload-containing fragments that localize the conjugation sites to heavy chain K395 and K417. UVPD yields an even higher sequence coverage (72%) with 28 one-payload- and 1 two-payload-containing fragment ions (Figure 3C). While EThcD and UVPD resulted in equivalent numbers of backbone cleavages between heavy chain K412 and K417, UVPD yielded greatly increased coverage between heavy chain K392 and K412, amplifying the confidence in site localization.

In the present strategy, acquisition of the HCD spectrum is the primary step used for the HCD-triggered methods, and thus this screening MS/MS spectrum was collected at a lower resolution and with a lower maximum ion injection time than the subsequent triggered EThcD and UVPD spectra. Characterization of the peptide by HCD is improved by increasing the resolution of the HCD scan, as shown in Figure S1 for the same 9.8 kDa peptide analyzed in Figure 3. The gain in sequence coverage using the higher resolution is minimal (increasing from 34% to 37%) and still fails to localize the payload site. The limitations of HCD in characterizing very large peptides have already been well established [36,42], and thus the optimal strategy utilizes the high speed and sensitivity of HCD (at lower spectral resolution) to generate reporter ions to trigger the subsequent slower but more informative EThcD or UVPD spectra (at higher spectral resolution). One additional benefit of the HCD screening step is the confidence gained by identifying the highly specific payload reporter ions which uniquely differentiate conjugated peptides from non-conjugated ones.

The complexity of the mass spectra increases with peptide size, necessitating the careful inspection of peptide spectral matches, particularly in cases where multiple lysine residues are present. To verify the quality of the data, a number of site-localizing fragment ions (ones which originate from backbone cleavages in regions of the sequence between lysine residues) were scrutinized for an exemplative peptide to ensure that their isotope profiles matched the theoretical composition of the assigned ions. Examples of isotopic fits for fragment ions bracketing the potential modified lysine sites of the peptide are shown in Figure S2. The isotopic fits shown in Figure S2B,C include examples of signal-to-noise and fit factors near the cutoff applied to all identified fragment ions. The validity of these threshold fragment ions demonstrates the quality of the data. Given the large number of peptides examined in this study, it is not feasible to manually validate all isotopic fits for all fragment ions for every peptide, as is often done in top-down studies that focus on localization of PTMs or in subunit-level middle-down studies of ADCs in which far fewer spectra are collected and curated [4,5]. As an additional criterion used in the present workflow, a payload site was considered unambiguous only upon detection of at least two fragment ions bracketing the modified lysine across multiple replicates. If two positional isomers were present, both isomers were considered to be confidently identified upon detection of at least four fragment ions unique to each position in at least three out of five replicates.

For peptides containing multiple lysine residues, peptides with different conjugation sites sometimes exhibited different elution times (see Figures S3 and S4) which facilitated the differentiation of conjugation sites. Other peptides were only observed as a single chromatographic peak which may or may not be composed of multiple conjugated isomers (Figure S5). Examination of the EThcD and UVPD fragmentation patterns in tandem was used to confirm the conjugation states. For each conjugated peptide, the sequence maps obtained by re-positioning the payload at each lysine were searched for diagnostic lysine-bracketing fragment ions. For example, in Figure S5, if the payload is positioned at heavy chain K65 for this 11.2 kDa peptide, then the sequence coverage is slightly lower than if the payload is positioned at heavy chain K76; however, there are many additional fragment ions generated by both EThcD and UVPD that support the localization of the payload to heavy chain K65. Therefore, it can be concluded that both conjugation states are likely present, and the peptides are not chromatographically resolved and so cannot be evaluated separately. The sequence maps for the 14.7 kDa peptides in Figure S3 have sufficient payload-localizing fragment ions to confidently pinpoint the conjugated lysine positions (light chain K45 and light chain K107). For the 12.3 kDa peptides in Figure S4, while the sequence map for the first peptide eluting at 32.40 min has adequate payload-localizing fragment ions to identify the payload at heavy chain K395, the second sequence map for the peptide eluting at 33.48 min does not. However, there are sufficient fragments in both the UVPD and EThcD spectra for the latter peptide to differentiate between payload conjugation at heavy chain K395 and heavy chain K412, indicating the presence of a secondary positional isomer with the payload at either heavy chain K412 or heavy chain K417.

### 3.2. Characterization of Heterogeneous Species with Multiple Conjugation Sites

**Figure 4:**
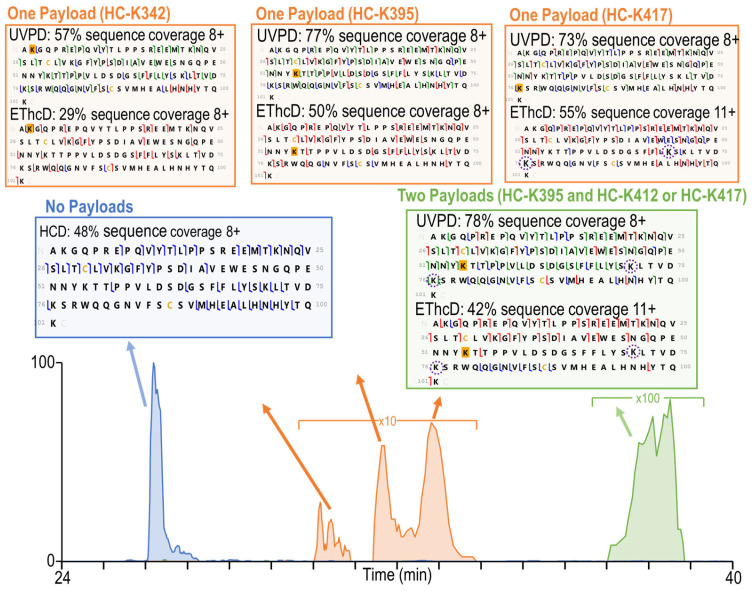
Extracted ion chromatograms (EICs) for a heavy chain peptide (101 amino acids, 8+) containing A342 through K442 with zero (*m*/*z* 1443.34) and one (*m*/*z* 1562.88) or two (*m*/*z* 1682.68) payloads. Sequence coverage maps are included for each chromatographic peak observed in the EIC. K2, K22, K32, K54, K71, and K76 of these sequences correspond to K342, K363, K373, K395, K412, and K417 of the heavy chain. Fragment ions discussed in the text are indicated with number labels. The payload localization sites are shaded in gold when unambiguous and circled when ambiguous. The chromatographic peaks are shaded to match the corresponding sequence maps.

While the peptides identified using ProSight PD, as listed in Tables S1–S3, only included peptides with single payload conjugations, the production of peptides containing multiple modified sites was also considered. Based on the typical average DAR of around 3.5 for T-DM1 [32] and the fact that the payloads may be located across four subunits (two heavy chains and two light chains), the probability that two payloads will be conjugated in sufficiently close proximity to result in doubly modified peptides is low when the ADCs are subjected to conventional bottom-up proteolytic methods. However, the large sizes of the peptides generated by limited proteolysis have the potential to allow for the detection and characterization of multiply conjugated species, although likely in low abundance. A total of seven peptides containing two payloads were identified by both UVPD and EThcD, as summarized in Tables S4 and S5. Given the increased complexity of these peptides, several examples are examined in more detail (Figure 4 and Figures S6–S8).

To identify and characterize peptides with multiple conjugated payloads, both the chromatographic data and the MS/MS spectra are examined. Figure 4 displays the extracted ion chromatogram for a peptide containing 101 amino acids with no payloads (blue trace), with one payload (orange trace), and with two payloads (green trace). The elution order of the three species offers one important feature. The payload is expected to increase the hydrophobicity of the peptide, resulting in a greater retention time as the number of payloads increases, as reflected in Figure 4. An HCD sequence coverage map is included for the peptide with no payloads. Given the high abundance and simplicity of this peptide, the HCD spectrum adequately characterizes it. Neither EThcD nor UVPD was triggered because the *m*/*z* 547.22 reporter ion was not generated by HCD. For the species with a single payload, three chromatographically resolved peaks were observed and attributed to three different conjugation states. Characterization of the peptide isomers containing a single conjugation at heavy chain K342 or heavy chain K395 was straightforward, with adequate diagnostic fragment ions generated by both UVPD and EThcD. Given the proximity of the two different lysine residues in this peptide (heavy chain residues K412 and K417), it is challenging to distinguish the exact conjugation sites for the third peptide isomer (t_r_ 33 min). The third peptide is well characterized by both UVPD and EThcD. In both cases, there are fragment ions originating from backbone cleavages between heavy chain K395 and heavy chain K412, indicating that the payload must be conjugated to heavy chain K412 or heavy chain K417. The site localization is particularly compelling based on the UVPD sequence coverage map for which there are more backbone cleavages between heavy chain K395 and heavy chain K412. With UVPD, there were also five fragment ions differentiating heavy chain K412 and heavy chain K417, indicating unambiguously and with high confidence that the payload is conjugate to heavy chain K417.

The heavy chain peptide with two payloads was examined. Based on the green chromatographic profile in Figure 4, the abundance of the peptide is low, diminishing the quality of the MS/MS spectra (and reducing the probability that the peptide would be targeted in any conventional data-dependent method). The doubly conjugated peptide was identified in all five replicates by both UVPD and EThcD based on automated ProSight PD analysis. In the sequence maps of the doubly conjugated peptide in Figure 4, the sequence coverage is sufficient to identify the peptide but is inadequate to completely localize the payloads. UVPD yielded a much higher sequence coverage of 78% compared to 42% obtained with EThcD, as well as a large number of fragment ions originated from backbone cleavages between heavy chain K373/K395 and heavy chain K395/K412, unambiguously localizing the first payload to heavy chain K395. UVPD also yielded two fragment ions produced from backbone cleavages between heavy chain K412 and heavy chain K417; however, while the *x*_30_ fragment ion contained a payload, the *x*_26_ fragment ion did not, leaving the conjugation site ambiguous based on the qualification metrics requiring two diagnostic fragment ions in order to unambiguously identify a conjugation site.

Figure 4 also demonstrates the power of the HCD-triggered method to optimize the data acquisition time for the most critical peptides. If only HCD spectra had been acquired to investigate the peptides, characterization of the sequence and payload sites would have been impeded. If only high-resolution EThcD or UVPD data had been collected, this peptide might have been missed because of its low abundance. By utilizing the HCD-triggered method which capitalizes on HCD as a screening tool and EThcD or UVPD for peptide characterization, even low-abundance two-payload-containing peptides are targeted for analysis.

Figures S6–S8 display the results for additional peptides for which two conjugation states (one payload or two payloads) were identified. In Figure S6, a shorter (24 amino acid) peptide was analyzed. While both conjugation isomers (light chain K188 and K190) containing one payload were identified by both UVPD and EThcD through ProSight PD analysis, the peptide with two payloads was only identified in triplicate by EThcD. Although the sequence coverage was rather low (35%) for this peptide, its validity was enhanced by the high-confidence identification of two structural isomers of the same peptide containing a single payload, confirming that both lysine residues can be targeted for conjugation. Figure S7 showcases a 65-residue peptide containing two lysine residues as likely conjugation sites as well as the heavy chain N-terminus as a third potential conjugation site. Sequence coverage by both UVPD and EThcD was adequate to localize the conjugation sites to heavy chain K43 and K30 for the peptides containing one or two payloads. Figure S8 displays results for a 66-residue peptide containing two proximal lysine residues (heavy chain K249 and K251). Only a single chromatographic peak is found for the peptide with one payload, but there is MS/MS evidence of two co-eluting positional isomers corresponding to the payload conjugated at heavy chain K251 or K277, each with sufficient fragment ions to differentiate heavy chain K249 and K251 based on both UVPD and EThcD spectra. Additionally, two fragment ions were observed in the UVPD spectrum for the peptide containing two payloads (*a_2_*_4_ and *y*_28_), differentiating the heavy chain K249 and K251 sites and allowing for the localization of the two payloads to heavy chain K241 and K277. The interpretation of these more complex peptides containing multiple payloads was only possible owing to the use of limited Lys-C digestion and the integration of enhanced tandem mass spectrometry methods.

### 3.3. Complete Characterization of Payload Binding Sites

**Figure 5:**
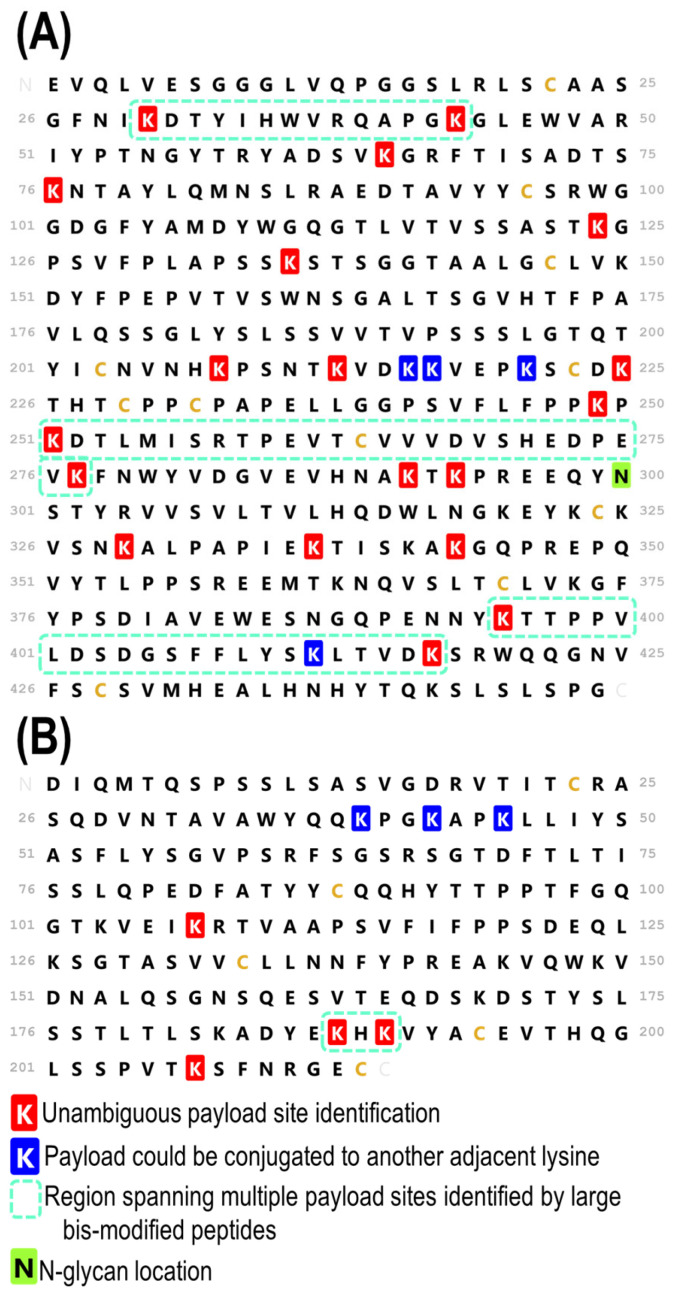
Sequence maps of the (**A**) HC and (**B**) LC of T-DM1 displaying the locations of the payloads, including those that were unambiguously localized (shaded in red) and those that remain ambiguous with adjacent or nearby lysine residues (shaded in blue). Regions containing multiple payload sites that are identified by large bis-conjugated peptides are outlined in dashed boxes.

In summary, the high-quality identification of peptides across the entire antibody sequence was achieved via a middle-down HCD-triggered MS/MS strategy. Figure 5 displays the comprehensive map of all the confirmed payload sites, including 4 unambiguous light chain sites, 19 unambiguous heavy chain sites, 3 ambiguous light chain sites, and 4 ambiguous heavy chain sites in which the payload could only be localized to a span of adjacent lysine residues. Maps of the identified peptides are displayed in Figure S9 (heavy chain) and Figure S10 (light chain), helping to visualize the overlapping payload-containing peptides that corroborate the localizations of conjugation site. While the total number of conjugation sites identified was lower than the number mapped in a recent bottom-up study [30], the results here represent a step towards unraveling the heterogeneity of lysine-conjugated ADCs. By examining the payload conjugation sites in the context of larger peptides, an improved understanding of the most prevalent conjugation sites and interplay among those sites is obtained. The focus of this study has been the heavy chain, given the inherent increased complexity imparted by its size and the larger number of payload-containing peptides identified relative to the light chain. As displayed in Figure 5, four unambiguous payload conjugation sites were identified in the light chain. The peptides leading to these four site localizations on the light chain are listed in Tables S1 and S2. Additionally, one multiply conjugated species was identified in the light chain, as detailed in Figure S6. Figure 5 also highlights in dashed boxes the regions in which the middle-down HCD-triggered MS/MS method revealed that multiple payloads were conjugated to one peptide. By identifying and characterizing peptides containing multiple lysine conjugation sites, a better understanding of regions containing high levels of ADC conjugation that might cause changes to antibody structure and function is gained.

Conventional bottom-up methods report the identification of 38–54 conjugation sites on lysine-linked ADCs out of 92 possible sites based on injections of a few micrograms of digest, with one study reporting the identification of 82 sites [31,32,33,34]. There are 92 possible conjugation sites, including 31 and 13 lysine residues on each heavy chain and light chain, respectively, plus an additional conjugation site at each N-terminal amine group (a total of 4). Out of those 92 conjugation sites, 46 were unambiguously identified based on the present middle-down approach, making these results comparable to those of most bottom-up studies. The ability to pinpoint locations where multiple payloads are conjugated in close proximity represents a level of information that has yet to be achieved by bottom-up or middle-down studies of ADCs. The information could be complemented by middle-up MS1 data generated from IdeS-digested ADCs [35,49], which would reveal the number of payloads bound to each subunit but not the locations of those payloads. While the limited digestion strategy demonstrated here represents one promising application of the HCD-triggered method for characterization of T-DM1, it could also be utilized to improve other middle-down, as well as bottom-up, workflows for T-DM1 and other ADCs that also generate signature fragment ions upon collisional activation and could benefit from a more targeted approach.

### 3.4. Comparison of a Second Batch of T-DM1

With the aim of validating the reproducibility of the methods presented here, a second batch of trastuzumab-emtansine was examined to evaluate broader feasibility. While the sample described in the majority of the text was in-date, the second sample was several years older and had been stored as a lyophilized powder and reconstituted for this experiment. The examination of this older sample allows for assessment of the integrity and variability of the ADC over time. The results for the second sample are summarized in Tables S6–S9. Additionally, sequence maps mirroring those in Figure 5 are shown in Figure S11, displaying the conjugation sites for the second sample. While there are slight differences in the maps, with one less unambiguous light chain identification as well as one missing and one additional unambiguous heavy chain identification for the second sample compared to the first, the findings are consistent overall. More broadly, there were fewer peptide identifications in the second sample and the sequence coverages tended to be lower, an outcome that could be attributed to the lower quality of the older sample. Overall, the peptides identified between the samples were primarily the same, including those identified with two payloads conjugated, validating both the reproducibility of the method and the integrity of the drug conjugation sites in the older sample.

### 3.5. Comparison to Bottom-Up Analysis

The same batches of trastuzumab-emtansine were subjected to trypsin proteolysis and nanoscale LC-MS/MS analysis to evaluate the outcomes obtained using this alternative bottom-up approach on the same mass spectrometer platform. Representative examples of the resulting base peak chromatograms are shown in Figure S12, and annotated sequence coverage maps displaying the identified payload-containing peptides based on EThcD and UVPD are shown in Figures S13 and S14, respectively. The locations of the payload-modified lysines found based on the bottom-up analysis are summarized in Figure S15. The numbers of peptide spectral matches obtained for payload-modified peptides ranged from 48 to 76 (see Table S10), and the numbers of payload-modified lysines ranged from 3 to 5 for the light chain and 12 to 15 for the heavy chain depending on the MS/MS method and batch (Table S11). These results are similar to those noted in another study that used a nanoscale LC method [33]. Analysis of the ADC digest revealed that the payload-modified peptides had low abundances, resulting in generally lower quality MS/MS spectra using either EThcD or UVPD. In general, there were few fragment ions retaining the payload despite the fact that the precursor peptides contained the appropriate mass shift corresponding to the payload (+956 Da). Therefore, confident payload localization was not achieved using bottom-up analysis, and overall fewer payload conjugation sites were identified compared to middle-down analysis. It was postulated that the ADCs (either the antibody or payload portions) might have degraded during the course of the study, but examination of the stored T-DM1 samples revealed good integrity based on the high-accuracy mass measurements of the intact ADCs infused directly (Figure S16). Moreover, sequence coverages for the antibody portion of the ADC ranged from 87% to 99% depending on the LC-MS/MS replicate, confirming the success of the trypsin proteolysis and MS/MS analysis (Figure S17). We speculate that the low abundances of the payload-containing peptides, in part owing to the low injection quantities on the nanoscale LC system and the substantially greater percentage of unmodified peptides, accounts for the rather low number of payload sites identified in this bottom-up analysis.

## 4. Discussion

The HCD-triggered MS/MS methods presented here represent a promising strategy for the characterization of highly heterogeneous lysine-linked ADCs. By applying an HCD-triggered method to large peptides generated from a middle-down proteolysis method, high-quality data were generated for large payload-containing peptides. The combination of results from EThcD and UVPD was particularly helpful for characterization of large peptides containing multiple potential payload sites; in some cases, one MS/MS method outperformed the other and in other cases only the combination of EThcD and UVPD yielded sufficient confidence to localize payloads. The number and abundances of peptides containing two payloads are low for the ADCs analyzed in the present study. This outcome is not unsurprising given that the expected drug-to-antibody ratio is approximately 3.5, meaning that on average there are three or four drugs conjugated to each antibody containing 92 potential conjugation sites. This means that the statistical likelihood of any peptide containing two or more payloads is low, and their anticipated low abundances makes them even more challenging to identify in heterogeneous mixtures containing numerous peptides.

The ability to estimate the conjugation efficiency of each lysine residue based on the current strategy is yet untested. Although one might be tempted to infer conjugation efficiencies based on peptide spectrum matches (i.e., a count based on the number of times each identified peptide is sampled) or based on the areas of the chromatographic peaks, these options have not been evaluated and need much deeper scrutiny. Moreover, the ability to correlate the specific conjugation site of a payload with its therapeutic efficacy is yet unresolved.

## 5. Conclusions

A key finding of the present study was that higher sequence coverage for large peptides did not always translate to complete localization of payloads, as localization often hinged on key regions between adjacent lysine residues. The generation of payload-containing fragment ions by EThcD and UVPD often proved critical to comprehensive characterization. Overall, 46 sites out of 92 were unambiguously identified through the characterization of 53 single-payload-containing peptides of varying molecular sizes. Seven peptides containing multiple conjugations were identified. The ability to identify multiply conjugated species, as enabled by these methods, proved crucial for deciphering heterogeneous lysine-linked ADCs and offers a compelling approach for more detailed characterization of biotherapeutics. The types of MS/MS methods described here are available on commercial mass spectrometers and thus should be accessible to biopharma companies.

It is well known that conjugation of lysine side chains results in heterogeneous ADCs, varying in both the number and sites of the attached payloads and resulting in potential variation in the therapeutic efficacy of each individual ADC molecule. Designing strategies to create more homogeneous ADCs with higher batch-to-batch consistency is an ongoing goal in the ADC manufacturing process and highlights the importance of simultaneously developing new analytical methods to characterize ADCs and monitor their structural changes over time and after administration.

## Data Availability

All data files are available in massive database (massive.ucsd.edu) with accession number MSV000092585. For access use username: MSV000092585_reviewer and password: ECWNov22.

## Supplementary Materials

The following supporting information can be downloaded at https://www.mdpi.com/article/10.3390/antib13020030/s1, Scheme S1: Structure of DM1 payload conjugated to trastuzumab and possible fragment ion structures that correspond to payload reporter ions of *m*/*z* 547.22, 485.22, and 453.19 Da observed in the HCD mass spectra; Figure S1: Sequence coverage maps of an 9.8 kDa peptide (6+) for HCD with (A) 30,000 and (B) 240,000 resolution at *m*/*z* 200. K22 and K44 of the peptide sequence shown here corresponds to K395 and K417 of the antibody heavy chain. The modified lysine sites are shaded in gold; Figure S2: Expanded regions of the UVPD mass spectrum shown in Figure 3C illustrating examples of fragment ion isotope patterns with (A) high fit factors and signal to noise, (B) low signal to noise, and (C) low fit factors. (D) Sequence map highlighting the backbone cleavage sites from which the identified fragment ions originated. In this and other sequence maps, backbone cleavages that lead to a/x (green), b/y (blue) and c/z (green) ions are overlaid, partially obscuring the color coded cleavage sites. The modified lysine sites are shaded in gold; Figure S3: Extracted ion chromatogram revealing two light chain peptides (14.7 kDa, 8+), each containing 126 residues (D1 through K126) and a single payload. Sequence coverage maps obtained by EThcD and UVPD localize the payload to K45 (peptide at 33.26 min) or K107 (peptide at 34.06 min). The payload localization sites are shaded in gold when unambiguous and circled when ambiguous; Figure S4: Extracted ion chromatogram revealing two heavy chain peptides (12.3 kDa, 8+), each containing 99 residues (G344 through K442) and a single payload. Sequence coverage maps obtained by EThcD and UVPD localize the payload to HC-K395 (peptide at 32.40 min) or HC-K412/K417 (peptide at 33.48 min). K52, K69, and K74 of the sequence correspond to K395, K412, and K417 of the heavy chain. The payload localization sites are shaded in gold when unambiguous and circled when ambiguous; Figure S5: Extracted ion chromatogram of a heavy chain peptide (11.2 kDa, 8+) containing G44 through K136 and a single payload. Sequence coverage maps are included for EThcD and UVPD which localize the payload to both K65 and K76, although they are not chromatically resolved and are therefore co-isolated. K22 and K33 of the sequence correspond to K65 and K76 of the heavy chain. The payload sites are shaded in gold when unambiguously localized; Figure S6: EICs for the 4+ charge state of a 24 amino acid long light chain peptide containing A184 through K207 with zero (*m*/*z* 673.33), one (*m*/*z* 913. 18) or two (*m*/*z* 1152.27) payload conjugations. Sequence coverage maps are included for each chromatographic peak observed in the EIC. K5 and K7 on these maps correspond to K188 and K190 on the light chain. The payload sites that are unambiguously localized are shaded in gold; Figure S7: EICs for the 6+ charge state of a 65 amino acid long heavy chain peptide containing E1 through K65 with zero (*m*/*z* 1179.11), one (*m*/*z* 1383.51) or two (*m*/*z* 1497.90) payload conjugations. Sequence coverage maps are included for each chromatographic peak observed in the EIC. The payload sites that are unambiguously localized are shaded in gold; Figure S8: EICs for the 7+ charge state of a 66 amino acid long heavy chain peptide containing T226 through K291 with zero (*m*/*z* 1039.81), one (*m*/*z* 1176. 58) or two (*m*/*z* 1313.34) payload conjugations. Sequence coverage map are included for each chromatographic peak observed in the EIC. The payload localization sites are shaded in gold when unambiguous and circled when ambiguous. K24, K26, and K52 on these maps correspond to K249, K251, and K277 on the heavy chain. Owing to co-elution, two maps are included for peptide corresponding to the light chain containing one payload (t_r_ 35 min); Figure S9: Peptide map displaying the global sequence coverage for the heavy chain. The same legend used in Figure 5 was retained for the heavy chain sequence. Peptides displayed in orange contain a single payload conjugation and peptides displayed in green contain two payload conjugations. Unambiguously localized payload conjugations sites are shown in black on for each peptide, and payloads that could be localized two multiple sites are shown in gray; Figure S10: Peptide map displaying the global sequence coverage for the light chain. The same legend used in Figure 5 was retained for the heavy chain sequence. Peptides displayed in orange contain a single payload conjugation. Unambiguously localized payload conjugations sites are shown in black on for each peptide, and payloads that could be localized two multiple sites are shown in gray. Figure S11: Sequence maps of the (A) heavy chain and (B) light chain of T-DM1 displaying the locations of the payloads, including those that were unambiguously localized (shared is red) and those that remain ambiguous with adjacent or nearby lysine residues (shaded in blue). Visualization displays results for secondary T-DM1 sample. Regions containing multiple payload sites that are identified by large bis-conjugated peptides are outlined in dashed boxes. Figure S12: Base peak chromatograms of T-DM1 from two batches ((A) is Lot 1 and (B) is Lot 2) subjected to reduction, alkylation, and trypsin digestion separated using a 62 min gradient. Peaks are labeled with base peak *m*/*z* and retention times and represent unmodified peptides. Payload-containing peptides are at least 2 orders of magnitude lower and are not labeled. Figure S13: Sequence coverage maps of the heavy chain and light chain of TDM-1 based on LC-MS/MS (UVPD) analysis of a tryptic digest. Only payload-containing peptides are shown in the maps, and these peptides are indicated by horizontal green bars. All lysines are labelled with red stars. All payload-modified lysines are outlined with black boxes. Fourteen payload-modified lysines were identified for the heavy chain, and four payload-modified peptides were identified for the light chain. Figure S14: Sequence coverage maps of the heavy chain and light chain of TDM-1 based on LC-MS/MS (EThcD) analysis of a tryptic digest. Only payload-containing peptides are shown in the maps, and these peptides are indicated by horizontal green bars. All lysines are labelled with red stars. All payload-modified lysines are outlined with black boxes. Twelve payload-modified lysines were identified for the heavy chain, and fivepayload-modified peptides were identified for the light chain. Figure S15: Sequence maps of the (A) heavy chain and (B) light chain of T-DM1 displaying the locations of the payloads identified using bottom-up analysis. Results from both batches of ADCs using both HCD-triggered EThcD and UVPD are combined. Modified lysines are shaded in gold. Figure S16: Mass spectrum of intact TDM-1 (lower) and deconvoluted mass spectrum (upper). The peaks are labelled with the number of attached payloads (P) in the deconvoluted mass spectrum. Figure S17: Peptide maps of the (A) heavy chain and (B) light chain of TDM-1 based on all identified peptides from the tryptic digest. All lysines are labelled with red stars. Lysines that are found to contain payloads are underlined with red dashes in the peptide sequences, all of which are marked with horizontal green bars. Table S1: List of single payload-containing peptides identified with ProSight PD for UVPD replicates in primary sample. For each peptide, the residue to which the payload was localized, the theoretical mass, as well as the sequence coverage and retention time for each replicate are listed. Some replicate entries are blank in the case that a peptide was not identified in all five technical replicates. Table S2: List of single payload-containing peptides identified with ProSight PD for EThcD replicates in primary sample. For each peptide, the residue to which the payload was localized, the theoretical mass, as well as the sequence coverage and retention time for each replicate are listed. Some replicate entries are blank in the case that a peptide was not identified in all five technical replicates. Table S3: Mass errors of identified fragment ions of the peptides listed in Tables S1 and S2. Table S4: List of two payload-containing peptides identified with ProSight PD for UVPD replicates in primary sample. For each peptide, the residue to which the payload was localized, the theoretical mass, as well as the sequence coverage and retention time for each replicate are listed. Some replicate entries are blank in the case that a peptide was not identified in all five technical replicates. Table S5: List of two payload-containing peptides identified with ProSight PD for EThcD replicates in primary sample. For each peptide, the residue to which the payload was localized, the theoretical mass, as well as the sequence coverage and retention time for each replicate are listed. Some replicate entries are blank in the case that a peptide was not identified in all five technical replicates. Table S6: List of single payload-containing peptides identified with ProSight PD for UVPD replicates in secondary sample. For each peptide, the residue to which the payload was localized, the theoretical mass, as well as the sequence coverage and retention time for each replicate are listed. Some replicate entries are blank in the case that a peptide was not identified in all five technical replicates. Table S7: List of single payload-containing peptides identified with ProSight PD for EThcD replicates in secondary sample. For each peptide, the residue to which the payload was localized, the theoretical mass, as well as the sequence coverage and retention time for each replicate are listed. Some replicate entries are blank in the case that a peptide was not identified in all five technical replicates. Table S8: List of two payload-containing peptides identified with ProSight PD for UVPD replicates in secondary sample. For each peptide, the residue to which the payload was localized, the theoretical mass, as well as the sequence coverage and retention time for each replicate are listed. Some replicate entries are blank in the case that a peptide was not identified in all five technical replicates. Table S9: List of two payload-containing peptides identified with ProSight PD for EThcD replicates in secondary sample. For each peptide, the residue to which the payload was localized, the theoretical mass, as well as the sequence coverage and retention time for each replicate are listed. Some replicate entries are blank in the case that a peptide was not identified in all five technical replicates. Table S10: Number of identified payload-modified peptide spectral matches for two different lots of TDM-1 based on bottom-up analysis of tryptic digests. Table S11: Number of payload-modified lysines identified in heavy and light chains (HC and LC) of two different lots of TDM-1 based on bottom-up analysis of tryptic digests. Structures of DM1-related fragments, HCD sequence coverage plots at low and high resolutions, expanded *m*/*z* regions to demonstrate quality of top-down data, extracted ion chromatogram and sequence coverage plots of LC peptides with one payload containing D1 through K126, extracted ion chromatogram and sequence coverage plots of heavy chain peptides with one payload containing G344 through K442, extracted ion chromatogram and sequence coverage plots of HC peptides with one payload containing G44 through K136, extracted ion chromatograms and sequence coverage plots of light chain peptides with 0–2 payloads containing A184 through K207, extracted ion chromatograms and sequence coverage plots of heavy chain peptides with 0–2 payloads containing E1 through K65, extracted ion chromatograms and sequence coverage plots of heavy chain peptides with 0–2 payloads containing T226 through K291, peptide map displaying the global sequence coverage on heavy chain, peptide map displaying the global sequence coverage on light chain, lists of payload-containing peptides identified from UVPD data, and lists of payload-containing peptides identified from EThcD data. (PDF) Tables displaying mass errors of identified fragment ions, including one example for each peptide from Tables S1 and S2 (Excel).
