# Supplementary material for: Enhanced Characterization of Lysine-Linked Antibody Drug Conjugates Enabled by Middle-Down Mass Spectrometry and Higher-Energy Collisional Dissociation-Triggered Electron-Transfer/Higher-Energy Collisional Dissociation and Ultraviolet Photodissociation
Source: Antibodies (Basel). 2024 Apr 17;13(2):30. doi: 10.3390/antib13020030 (PMC11036284; doi:10.3390/antib13020030)
Supplement: Supplementary file 1 [file antibodies-13-00030-s001.zip › antibodies-2904107-supplementary/antibodies-2904107-supplementary.pdf]

Supporting Information

Enhanced Characterization of Lysine-Linked Antibody Drug Conjugates Enabled by Middle-Down Mass Spectrometry and HCD-Triggered EThcD and UVPD

Eleanor Watts[1], Aarti Bashyal,[1] Sean D. Dunham,[1] Christopher M. Crittenden[2], Jennifer S. Brodbelt[1]

[1]Department of Chemistry, University of Texas at Austin, Austin, TX 78712

[2] Small Molecule Pharmaceutical Sciences, Genentech Inc., South San Francisco, CA 94080
Correspondence to: jbrodbelt@cm.utexas.edu

Content	Content	Page No
Scheme S1	Structures of DM1 related fragments	S-3
Figure S1	HCD sequence coverage maps at low and high resolutions	S-4
Figure S2	Expanded m/z regions to demonstrate quality of top-down data	S-5
Figure S3	Extracted ion chromatogram and sequence coverage maps of LC peptides containing 126 residues (D1 through K126) and one payload	S-6
Figure S4	Extracted ion chromatogram and sequence coverage maps of HC peptides containing 99 residues (G344 through K442) and one payload	S-7
Figure S5	Extracted ion chromatogram and sequence coverage maps of HC peptides containing 93 residues (G44 through K136) and one payload	S-8
Figure S6	Extracted ion chromatograms and sequence coverage maps of LC peptides containing 24 residues (A184 through K207) and 0-2 payloads	S-9
Figure S7	Extracted ion chromatograms and sequence coverage maps of HC peptides containing 65 residues (E1 through K65) and 0-2 payloads	S-10
Figure S8	Extracted ion chromatograms and sequence coverage maps of HC peptides containing 66 residues (T226 through K291) and 0-2 payloads	S-11
Figure S9	Peptide map displaying the global sequence coverage of heavy chain	S-12
Figure S10	Peptide map displaying the global sequence coverage of light chain	S-13
Figure S11	Sequence maps displaying payload conjugation locations for secondary sample	S-14
Figure S12	Base peak chromatograms of tryptic digests of T-DM1 from two batches.	S-15
Figure S13	Sequence coverage maps of the heavy chain and light chain of TDM-1 based on LC-MS/MS (UVPD) analysis of a tryptic digest.	S-16

Figure S14	Sequence coverage maps of the heavy chain and light chain of TDM-1 based on LC-MS/MS (EThcD) analysis of a tryptic digest.	S-17
Figure S15	Sequence maps of the (A) heavy chain and (B) light chain of T-DM1 displaying the locations of the payloads identified using bottom-up analysis.	S-18
Figure S16	Mass spectrum of intact TDM-1 (lower) and deconvoluted mass spectrum (upper).	S-19
Figure S17	Peptide maps of the A) heavy chain and B) light chain of TDM-1 based on all identified peptides from the tryptic digest.	S-20
Table S1	List of single payload-containing peptides identified from UVPD data for primary sample	S-21
Table S2	List of single payload-containing peptides identified from EThcD data for primary sample	S-23
Table S3	Mass errors of identified fragment ions of the peptides listed in Tables S1 and S2	Excel
Table S4	List of two payload-containing peptides identified from UVPD data for primary sample	S-25
Table S5	List of two payload-containing peptides identified from EThcD data for primary sample	S-26
Table S6	List of single payload-containing peptides identified from UVPD data for secondary sample	S-27
Table S7	List of single payload-containing peptides identified from EThcD data for secondary sample	S-29
Table S8	List of two payload-containing peptides identified from UVPD data for secondary sample	S-31
Table S9	List of two payload-containing peptides identified from EThcD data for secondary sample	S-32
Table S10	Number of identified payload-modified peptide spectral matches for two different lots of TDM-1 based on bottom-up analysis of tryptic digests.	S-33
Table S11	Number of payload-modified lysines identified in heavy and light chains (HC and LC) of two different lots of TDM-1 based on bottom-up analysis of tryptic digests.	S-34

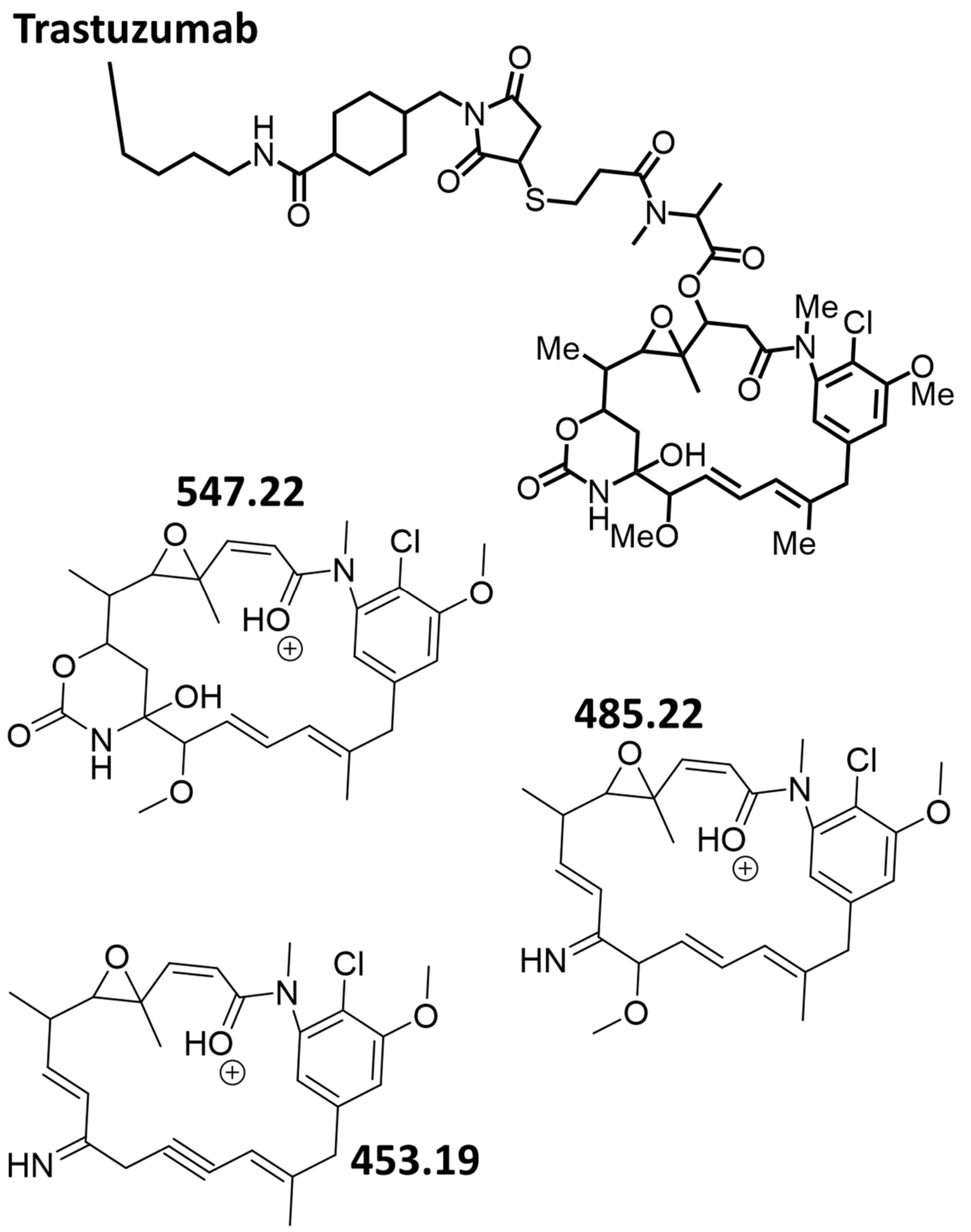

Scheme S1. Structure of DM1 payload conjugated to trastuzumab and possible fragment ion structures that correspond to payload reporter ions of *m*/*z* 547.22, 485.22, and 453.19 Da observed in the HCD mass spectra.

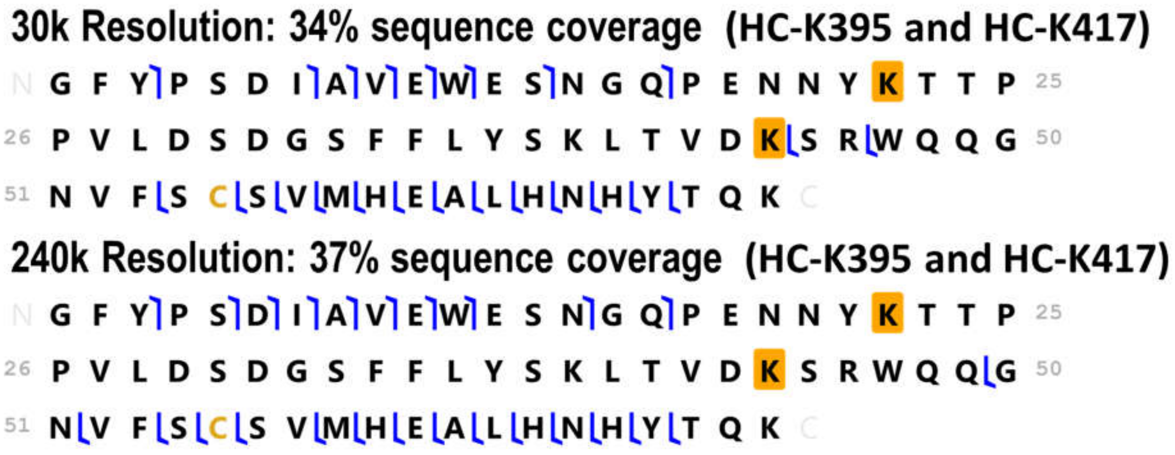

Figure S1. Sequence coverage maps of an 9.8 kDa peptide (6+) for HCD with (A) 30,000 and (B) 240,000 resolution at *m/z* 200. K22 and K44 of the peptide sequence shown here corresponds to K395 and K417 of the antibody heavy chain. The modified lysine sites are shaded in gold.

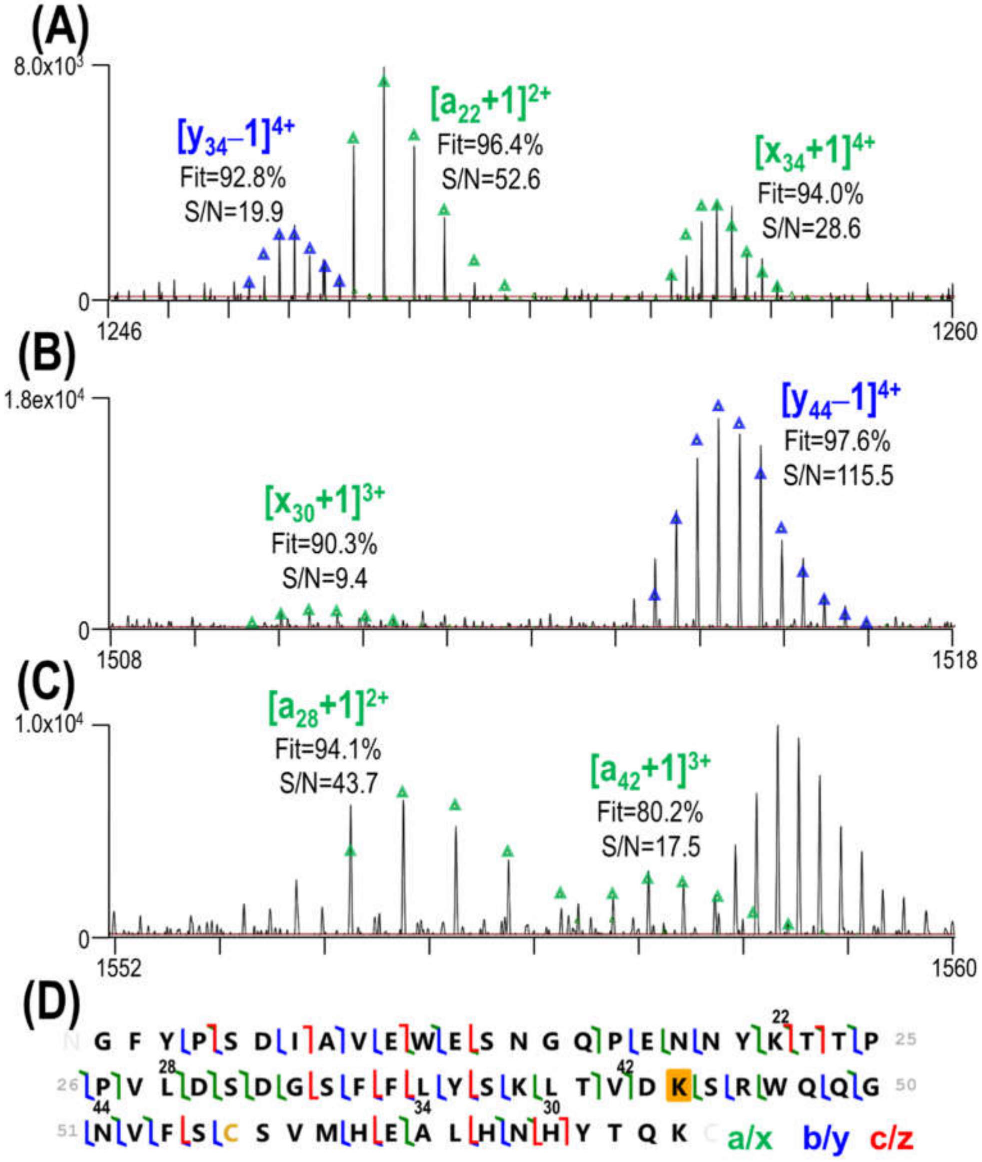

Figure S2. Expanded regions of the UVPD mass spectrum shown in **Figure 3C** illustrating examples of fragment ion isotope patterns with (A) high fit factors and signal to noise, (B) low signal to noise, and (C) low fit factors. (D) Sequence map highlighting the backbone cleavage sites from which the identified fragment ions originated. In this and other sequence maps, backbone cleavages that lead to a/x (green), b/y (blue) and c/z (green) ions are overlaid, partially obscuring the color coded cleavage sites. The modified lysine sites are shaded in gold.

A variety of fragment ions were identified with and without the payload, including C-terminal payload containing fragments (A) $[y_{34}\text{-}1]^{4+}$, (A) $[x_{34}+1]^{4+}$, (B) $[x_{30}+1]^{3+}$, and (B) $[y_{44}\text{-}1]^{4+}$, and N-terminal fragments without the payload (A) $[a_{22}+1]^{2+}$, (C) $[a_{28}\text{-}+]^{2+}$, and (C) $[a_{42}+1]^{3+}$. Fit factors are calculated in Thermo Xtract and signal to noise values and the theoretical isotopic distributions are generated with TDValidator.

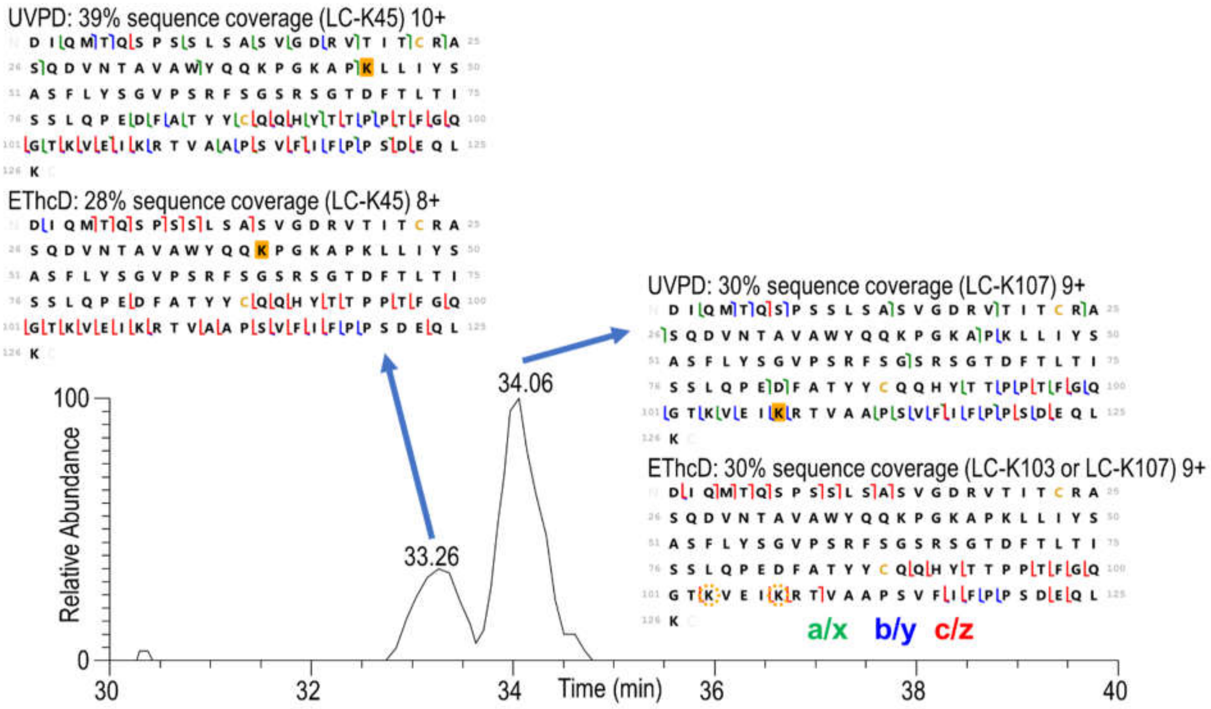

Figure S3. Extracted ion chromatogram revealing two light chain peptides (14.7 kDa, 8+), each containing 126 residues (D1 through K126) and a single payload. Sequence coverage maps obtained by EThcD and UVPD localize the payload to K45 (peptide at 33.26 min) or K107 (peptide at 34.06 min). The payload localization sites are shaded in gold when unambiguous and circled when ambiguous.

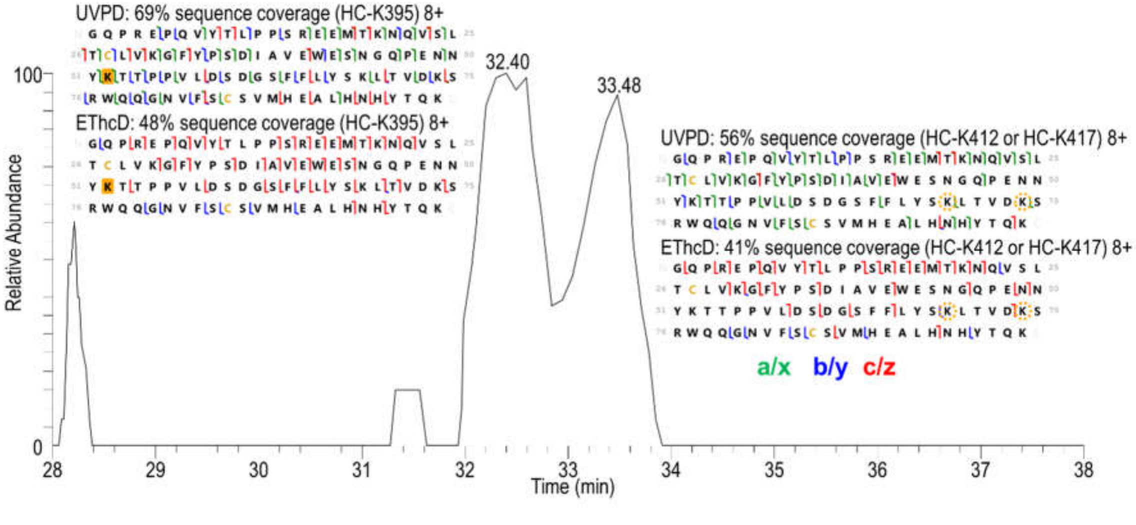

Figure S4. Extracted ion chromatogram revealing two heavy chain peptides (12.3 kDa, 8+), each containing 99 residues (G344 through K442) and a single payload. Sequence coverage maps

obtained by EThcD and UVPD localize the payload to HC-K395 (peptide at 32.40 min) or HC-K412/K417 (peptide at 33.48 min). K52, K69, and K74 of the sequence correspond to K395, K412, and K417 of the heavy chain. The payload localization sites are shaded in gold when unambiguous and circled when ambiguous.

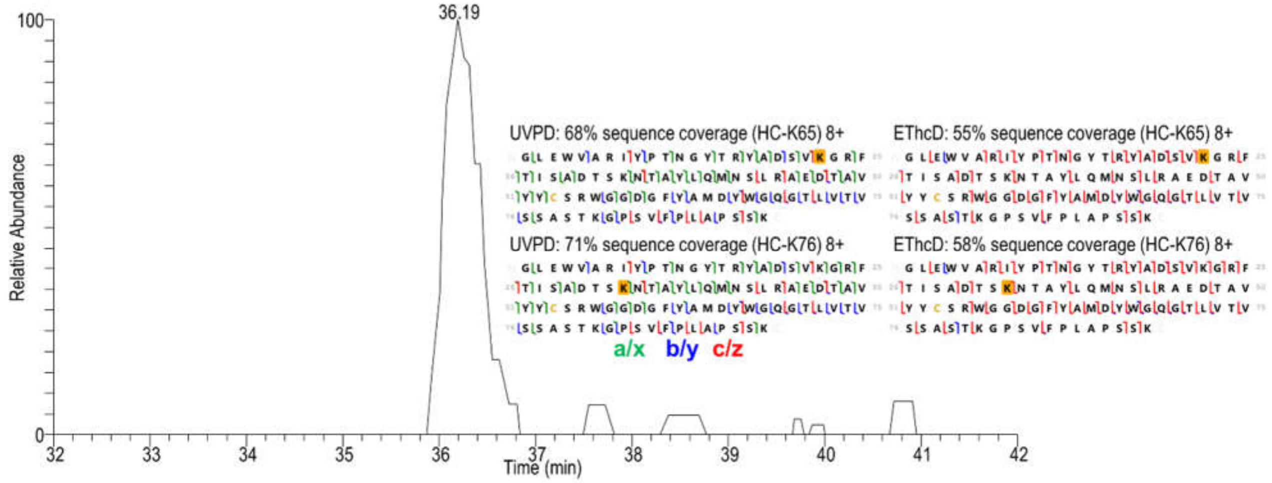

Figure S5. Extracted ion chromatogram of a heavy chain peptide (11.2 kDa, 8+) containing G44 through K136 and a single payload. Sequence coverage maps are included for EThcD and UVPD which localize the payload to both K65 and K76, although they are not chromatically resolved and are therefore co-isolated. K22 and K33 of the sequence correspond to K65 and K76 of the heavy chain. The payload sites are shaded in gold when unambiguously localized.

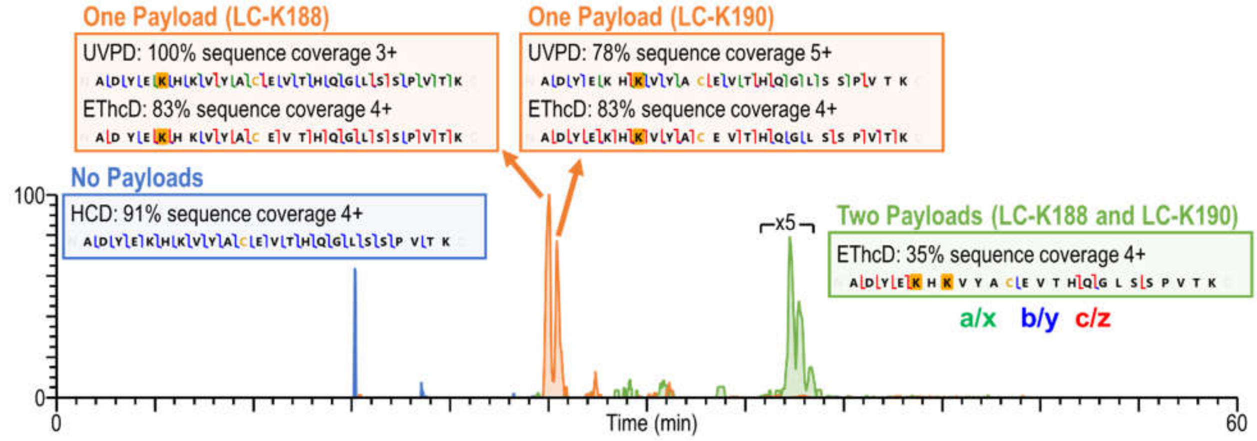

Figure S6. EICs for the 4+ charge state of a 24 amino acid long light chain peptide containing A184 through K207 with zero (*m/z* 673.33), one (*m/z* 913. 18) or two (*m/z* 1152.27) payload conjugations. Sequence coverage maps are included for each chromatographic peak observed in

the EIC. K5 and K7 on these maps correspond to K188 and K190 on the light chain. The payload sites that are unambiguously localized are shaded in gold.

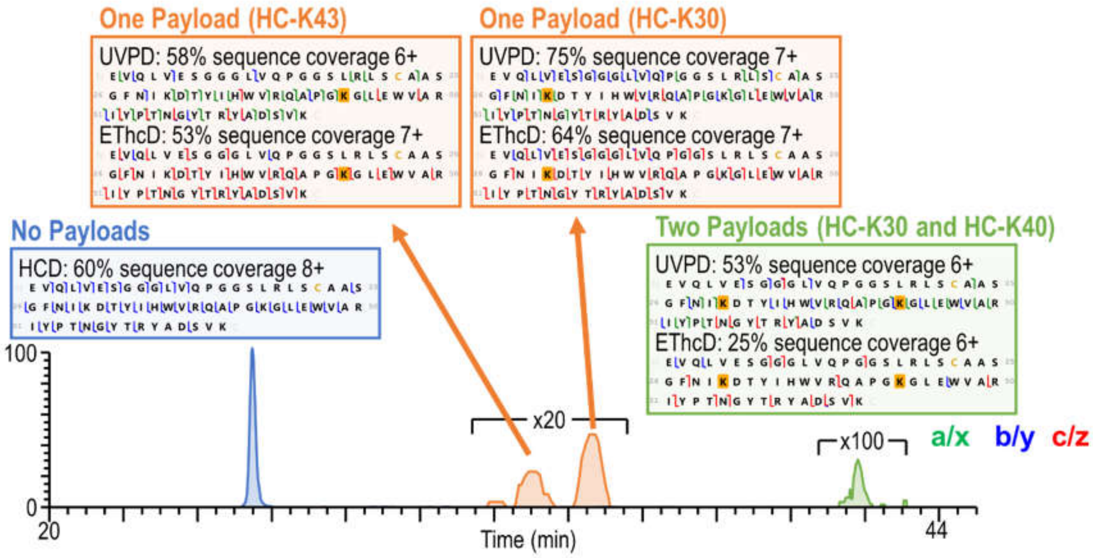

Figure S7. EICs for the 6+ charge state of a 65 amino acid long heavy chain peptide containing E1 through K65 with zero (*m/z* 1179.11), one (*m/z* 1383.51) or two (*m/z* 1497.90) payload conjugations. Sequence coverage maps are included for each chromatographic peak observed in the EIC. The payload sites that are unambiguously localized are shaded in gold.

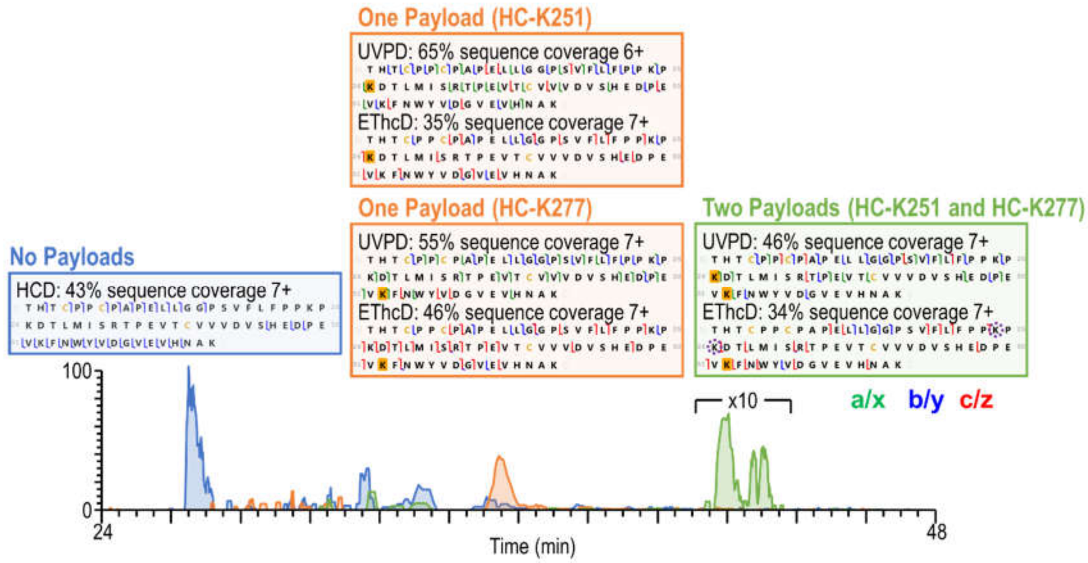

Figure S8. EICs for the 7+ charge state of a 66 amino acid long heavy chain peptide containing T226 through K291 with zero (*m/z* 1039.81), one (*m/z* 1176. 58) or two (*m/z* 1313.34) payload conjugations. Sequence coverage map are included for each chromatographic peak observed in the EIC. The payload localization sites are shaded in gold when unambiguous and circled when

ambiguous. K24, K26, and K52 on these maps correspond to K249, K251, and K277 on the heavy chain. Owing to co-elution, two maps are included for peptide corresponding to the light chain containing one payload (t_r 35 min).

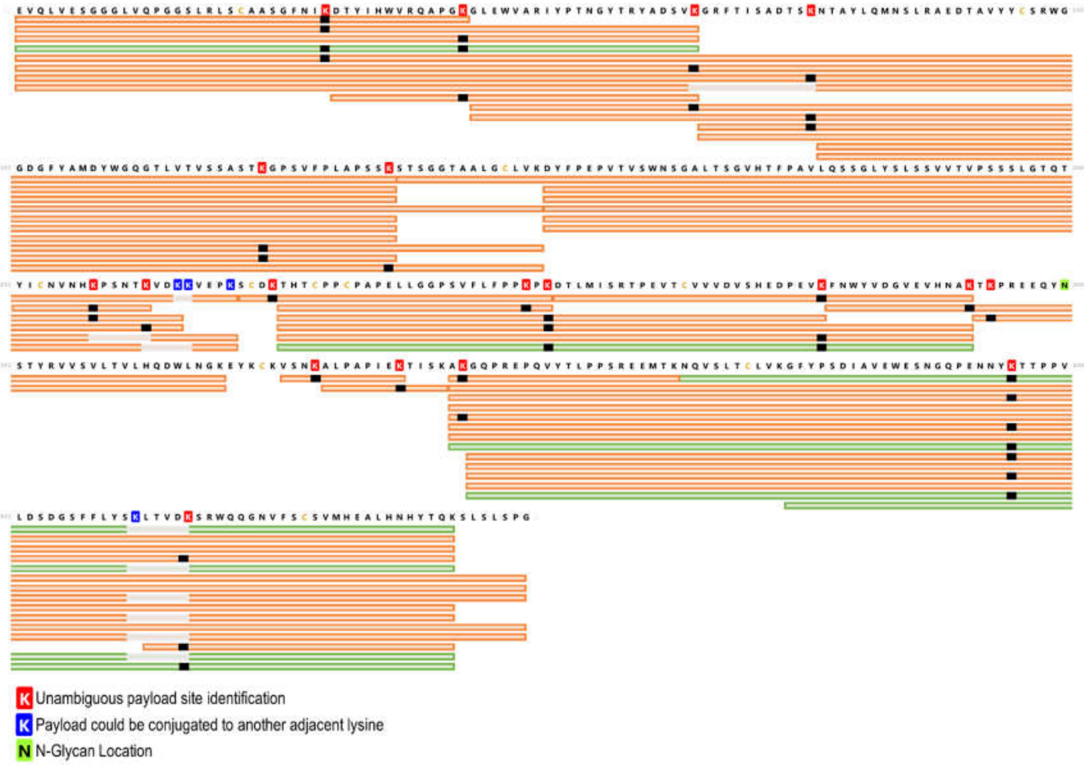

Figure S9. Peptide map displaying the global sequence coverage for the heavy chain. The same legend used in Figure 5 was retained for the heavy chain sequence. Peptides displayed in orange contain a single payload conjugation and peptides displayed in green contain two payload conjugations. Unambiguously localized payload conjugations sites are shown in black on for each peptide, and payloads that could be localized two multiple sites are shown in gray.

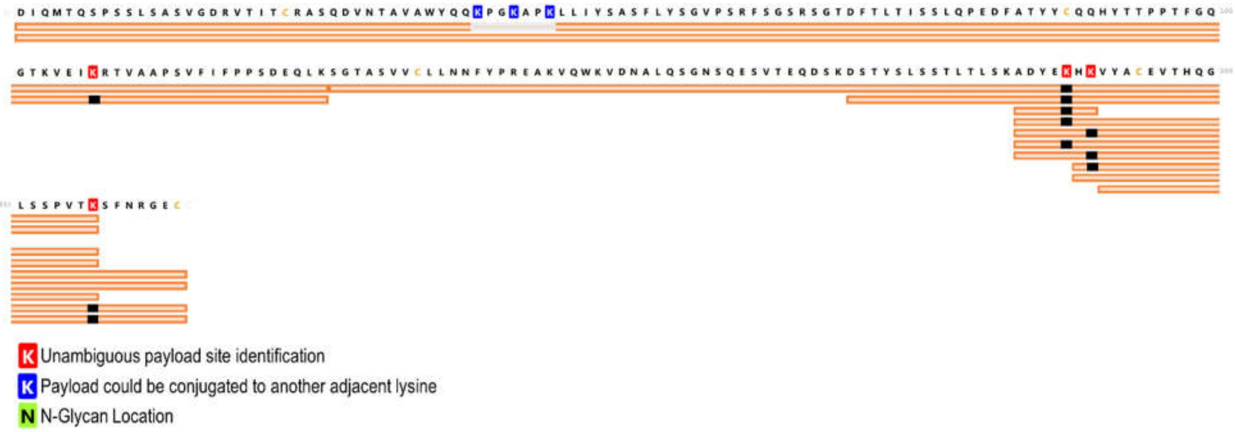

Figure S10. Peptide map displaying the global sequence coverage for the light chain. The same legend used in Figure 5 was retained for the heavy chain sequence. Peptides displayed in

orange contain a single payload conjugation. Unambiguously localized payload conjugations sites are shown in black on for each peptide, and payloads that could be localized two multiple sites are shown in gray.

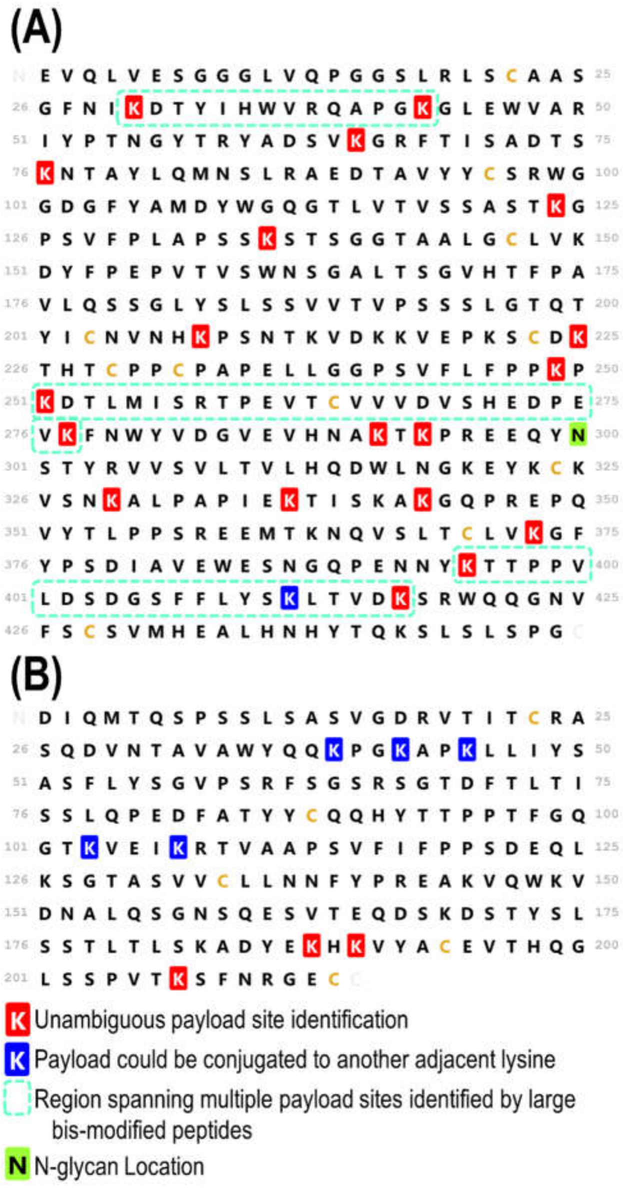

Figure S11. Sequence maps of the (A) heavy chain and (B) light chain of T-DM1 displaying the locations of the payloads, including those that were unambiguously localized (shared is red) and those that remain ambiguous with adjacent or nearby lysine residues (shaded in blue). Visualization displays results for secondary T-DM1 sample. Regions containing multiple payload sites that are identified by large bis-conjugated peptides are outlined in dashed boxes.

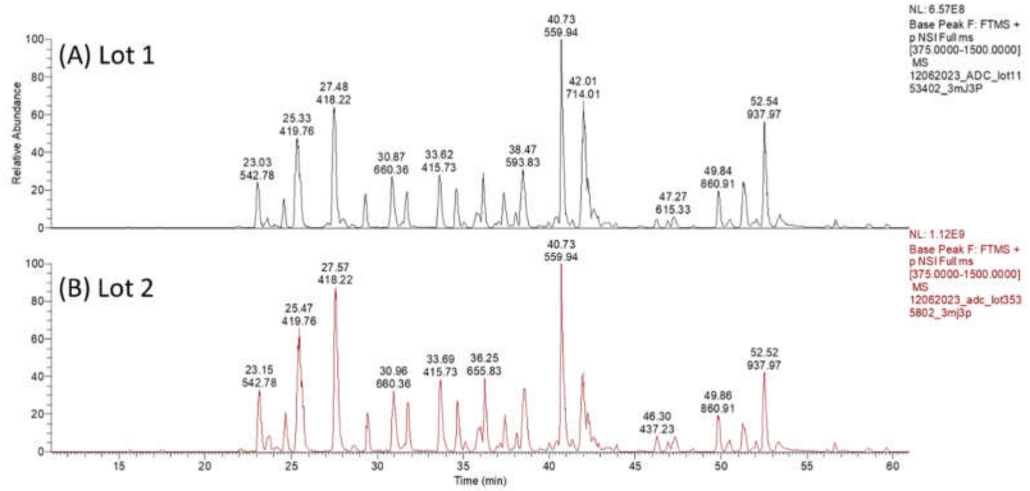

Figure S12. Base peak chromatograms of T-DM1 from two batches ((**A**) is Lot 1 and (**B**) is Lot 2) subjected to reduction, alkylation, and trypsin digestion separated using a 62 min gradient. Peaks are labeled with base peak m/z and retention times and represent unmodified peptides. Payload-containing peptides are at least 2 orders of magnitude lower and are not labeled.

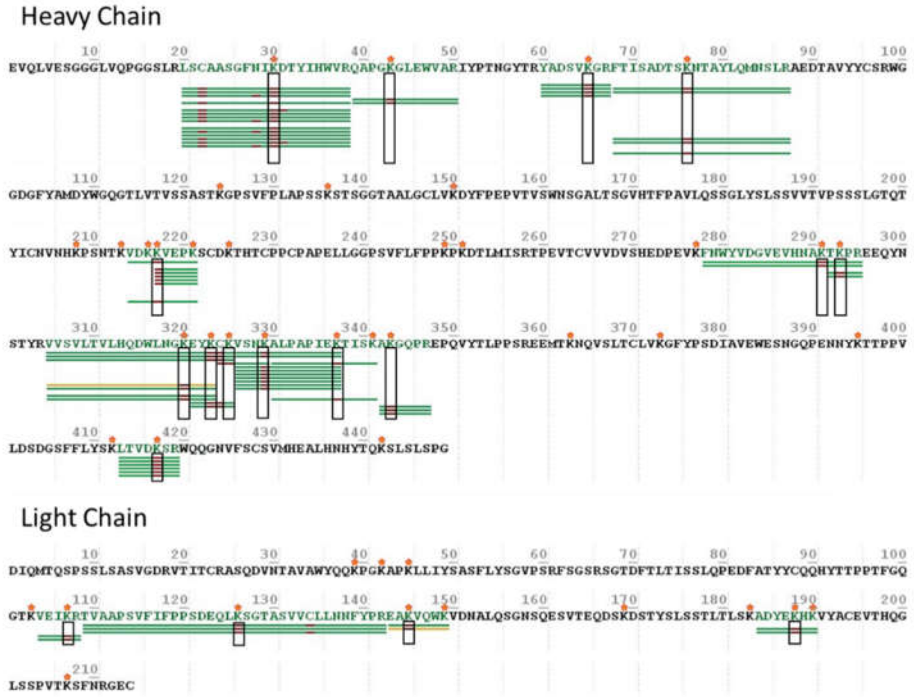

Figure S13: Sequence coverage maps of the heavy chain and light chain of TDM-1 based on LC-MS/MS (UVPD) analysis of a tryptic digest. Only payload-containing peptides

are shown in the maps, and these peptides are indicated by horizontal green bars. All lysines are labelled with red stars. All payload-modified lysines are outlined with black boxes. Fourteen payload-modified lysines were identified for the heavy chain, and four payload-modified peptides were identified for the light chain.

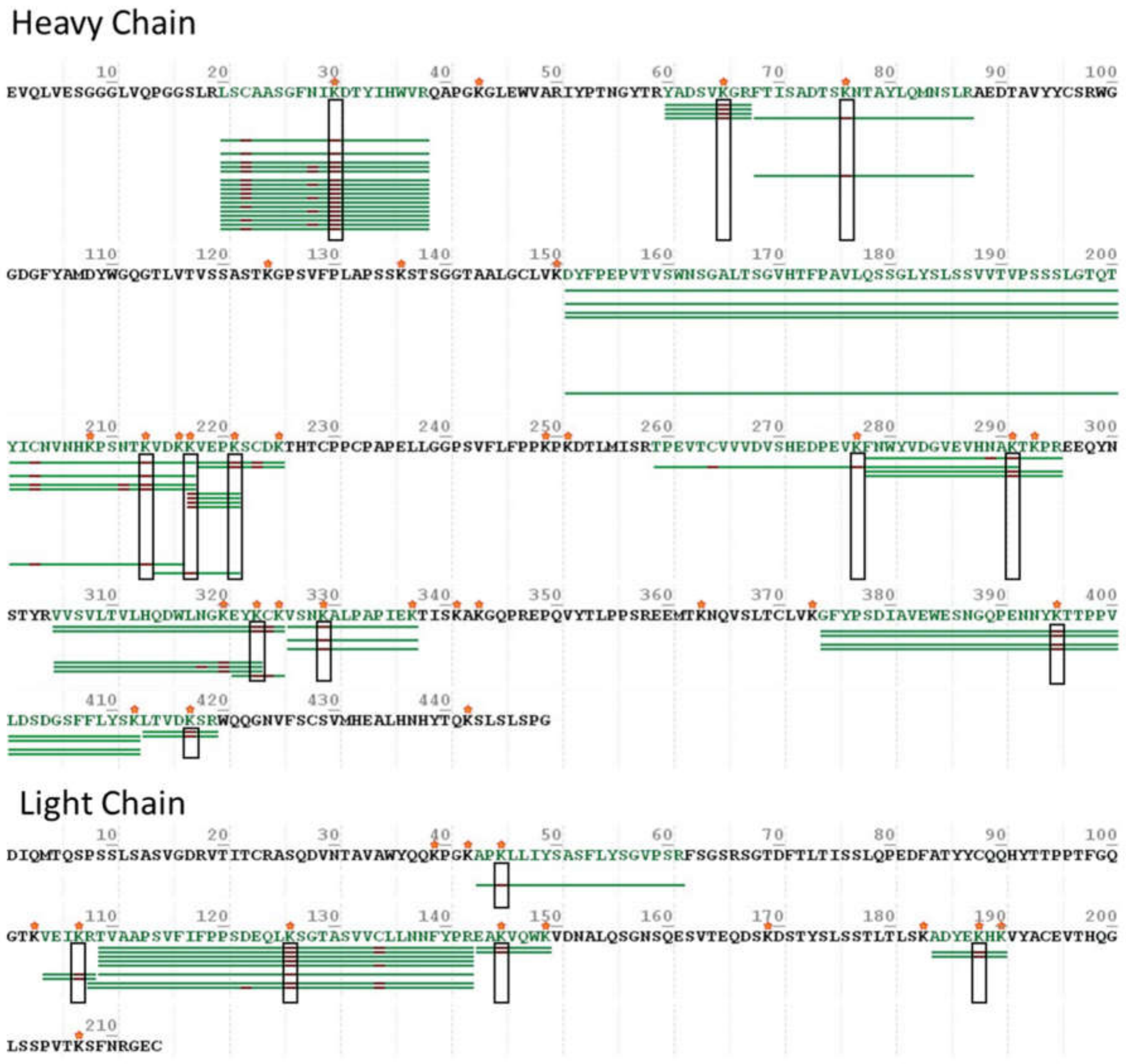

Figure S14. Sequence coverage maps of the heavy chain and light chain of TDM-1 based on LC-MS/MS (EThcD) analysis of a tryptic digest. Only payload-containing peptides are shown in the maps, and these peptides are indicated by horizontal green bars. All lysines are labelled with red stars. All payload-modified lysines are outlined with black boxes. Twelve payload-modified lysines were identified for the heavy chain, and fivepayload-modified peptides were identified for the light chain.

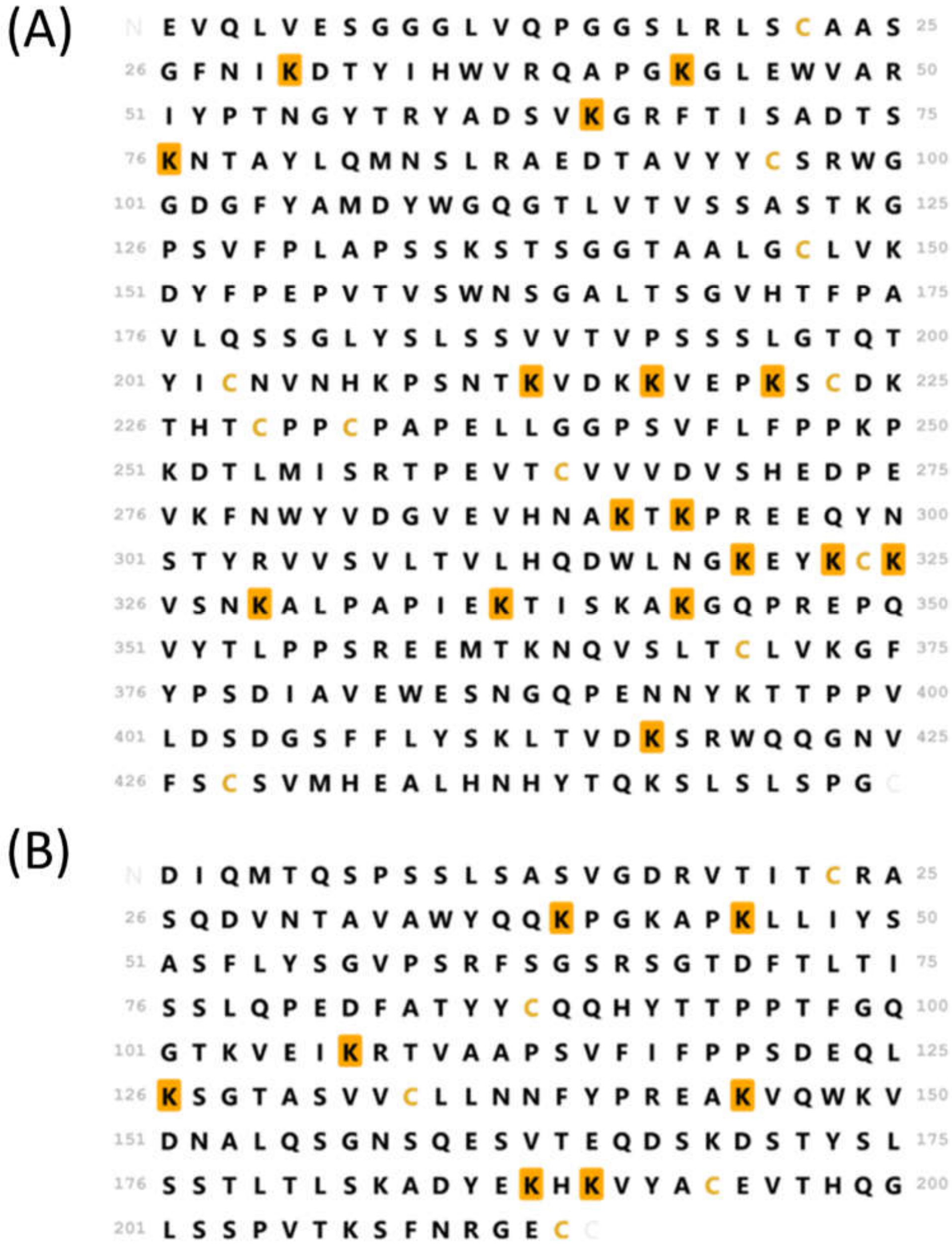

Figure S15. Sequence maps of the (A) heavy chain and (B) light chain of T-DM1 displaying the locations of the payloads identified using bottom-up analysis. Results from both batches of ADCs using both HCD-triggered EThcD and UVPD are combined. Modified lysines are shaded in gold.

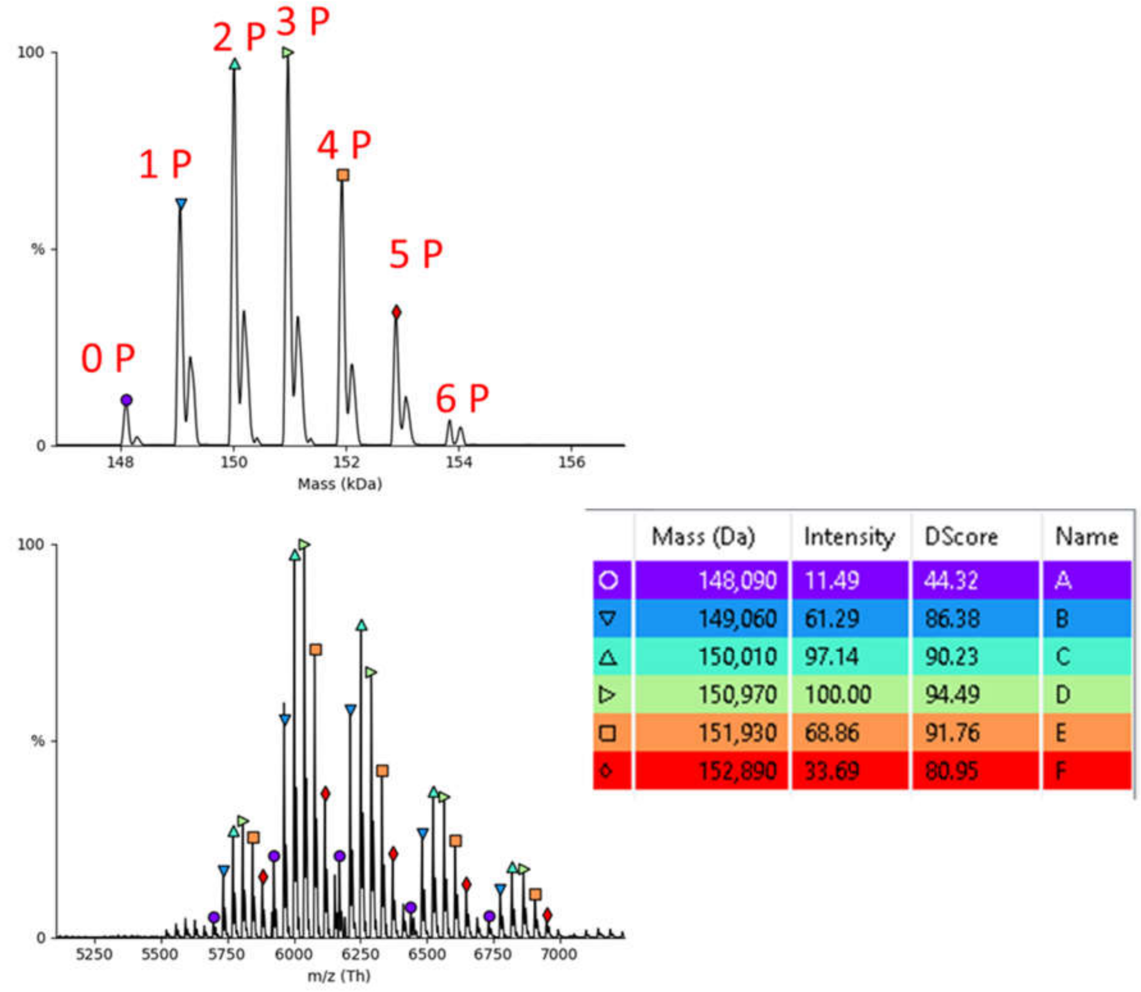

	Mass (Da)	Intensity	DScore	Name
○	148,090	11.49	44.32	A
▽	149,060	61.29	86.38	B
△	150,010	97.14	90.23	C
▷	150,970	100.00	94.49	D
□	151,930	68.86	91.76	E
◇	152,890	33.69	80.95	F

Figure S16. Mass spectrum of intact TDM-1 (lower) and deconvoluted mass spectrum (upper). The peaks are labelled with the number of attached payloads (P) in the deconvoluted mass spectrum.

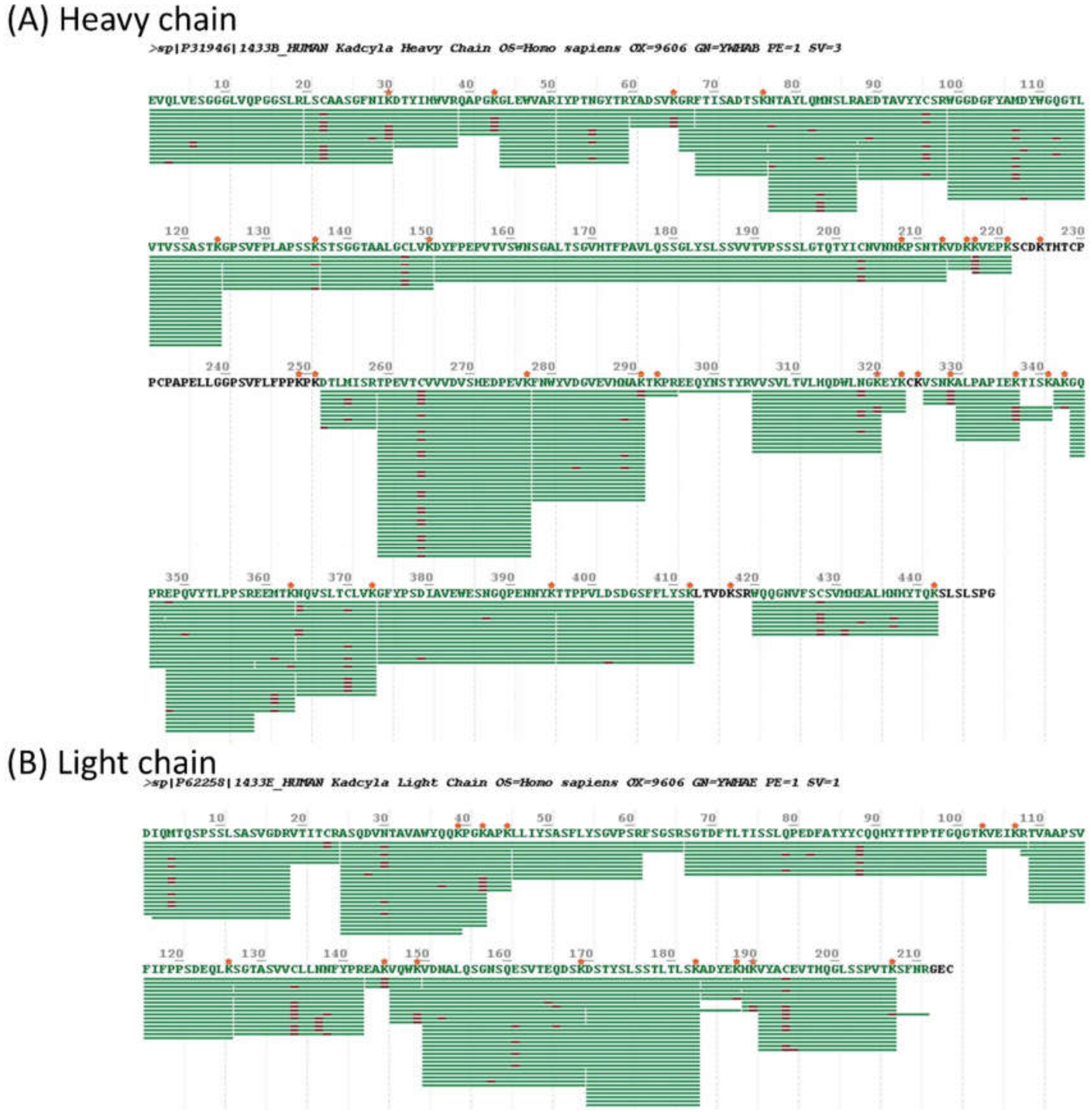

Figure S17. Peptide maps of the (**A**) heavy chain and (**B**) light chain of TDM-1 based on all identified peptides from the tryptic digest. All lysines are labelled with red stars. Lysines that are found to contain payloads are underlined with red dashes in the peptide sequences, all of which are marked with horizontal green bars.

Table S1. List of single payload-containing peptides identified with ProSight PD for UVPD replicates in primary sample. For each peptide, the residue to which the payload was localized, the theoretical mass, as well as the sequence coverage and retention time for each replicate are listed. Some replicate entries are blank in the case that a peptide was not identified in all five technical replicates.

Annotated Sequence	Payload Localization (residue number on peptide sequence)	Payload Localization (residue number on protein sequence)	Theo. Mass [Da]	Replicate 1		Replicate 2		Replicate 3		Replicate 4		Replicate 5	
				SC	RT	SC	RT	SC	RT	SC	RT	SC	RT
ADYEKHK	K5	LC-K188	1845.79	83%	26.1	83%	25.9	83%	26.0	83%	25.9	100%	26.2
ADYEKHKVYACEVTHQGLSSPVTK	K5	LC-K188	3645.68	96%	25.1	100%	24.9	96%	25.0	100%	24.9	96%	25.1
ADYEKHKVYACEVTHQGLSSPVTK	K7	LC-K190	3645.68	74%	25.8	70%	25.5			78%	25.6	52%	25.8
ADYEKHKVYACEVTHQGLSSPVTKSFNRGEC	K5	LC-K188	4439.00	90%	24.9	90%	24.8	93%	24.9	90%	24.8	90%	25.0
ADYEKHKVYACEVTHQGLSSPVTKSFNRGEC	K7	LC-K190	4439.00	67%	25.7	80%	25.5	73%	25.7	63%	25.4	63%	25.7
AKGQPREPQVYTLPPSREEMTKNQVSLTCLVKGFYPSDIAVEWESNGQPENNYKTTPPVLDSDGSFFLYSKLTVDKSRWQQGNVFSCSVMHEALHNHYTQK	K2	HC-K343	12487.96	56%	30.2	58%	30.2	43%	30.4	28%	29.9	35%	30.5
AKGQPREPQVYTLPPSREEMTKNQVSLTCLVKGFYPSDIAVEWESNGQPENNYKTTPPVLDSDGSFFLYSKLTVDKSRWQQGNVFSCSVMHEALHNHYTQK	K54	HC-K395	12487.96	71%	31.7	60%	31.4	68%	31.5	67%	31.4	77%	31.7
AKGQPREPQVYTLPPSREEMTKNQVSLTCLVKGFYPSDIAVEWESNGQPENNYKTTPPVLDSDGSFFLYSKLTVDKSRWQQGNVFSCSVMHEALHNHYTQK	K76	HC-K417	12487.96	58%	32.8	73%	32.4	67%	32.6	66%	32.5	67%	32.9
AKGQPREPQVYTLPPSREEMTKNQVSLTCLVKGFYPSDIAVEWESNGQPENNYKTTPPVLDSDGSFFLYSKLTVDKSRWQQGNVFSCSVMHEALHNHYTQKSLSLSPG	K2	HC-K343	13129.29	37%	30.4	28%	30.2	38%	30.4	52%	30.3	33%	30.7
AKGQPREPQVYTLPPSREEMTKNQVSLTCLVKGFYPSDIAVEWESNGQPENNYKTTPPVLDSDGSFFLYSKLTVDKSRWQQGNVFSCSVMHEALHNHYTQKSLSLSPG	K54	HC-K395	13129.29	65%	32.0	75%	31.6	62%	31.9	74%	31.6	74%	31.8
AKGQPREPQVYTLPPSREEMTKNQVSLTCLVKGFYPSDIAVEWESNGQPENNYKTTPPVLDSDGSFFLYSKLTVDKSRWQQGNVFSCSVMHEALHNHYTQKSLSLSPG	K71 or K76	HC-K412 or HC-K417	13129.29	61%	33.1	67%	32.9	63%	33.1	66%	33.0	59%	33.3
DIQMTQSPSSLSASVGDRVTITCRASQDVNTAVAWYQQKPGKAPKLLIYSASFLYSGVPSRFSGSRSGTDFTLTISSLQPEDFATYYCQQHYTTPPTFGQGTKVEIKRTVAAPSVFIFPPSDEQLK	K103 or K107	LC-K103 or LC-K107	14728.24	23%	33.3	30%	33.0	30%	33.0	22%	33.2	23%	33.4
DIQMTQSPSSLSASVGDRVTITCRASQDVNTAVAWYQQKPGKAPKLLIYSASFLYSGVPSRFSGSRSGTDFTLTISSLQPEDFATYYCQQHYTTPPTFGQGTKVEIKRTVAAPSVFIFPPSDEQLK	K39, K42, or K45	LC-K39, LC-K42, or LC-K45	14728.24	39%	34.2	36%	33.8	35%	33.9	35%	33.8	39%	34.0
DSTYSLSSTLTLSKADYEKHKVYACEVTHQGLSSPVTK	K19	LC-K188	5129.42	54%	28.1	32%	27.8	43%	28.0	35%	27.9	41%	28.2
DTLMISRTPEVTCVVVDVSHEDPEVKFNWYVDGVEVHNAK	K26	HC-K277	5512.56	62%	35.6	67%	35.4	74%	35.5	56%	35.5	69%	35.6
DTYIHWVRQAPGKGLEWVARIYPTNGYTRYADSVK	K13	HC-K43	5066.45	29%	31.5	44%	31.2			24%	31.2	50%	31.5
DYFPEPVTVSWNSGALTSGVHTFPAVLQSSGLYSLSSVVTVPSSSLGTQTYICNVNHKPSNTK	K58	HC-K208	7611.65	40%	36.0	34%	35.9	32%	36.3	39%	35.9	23%	36.1
DYFPEPVTVSWNSGALTSGVHTFPAVLQSSGLYSLSSVVTVPSSSLGTQTYICNVNHKPSNTKVDK	K63	HC-K213	7953.84	46%	34.5	45%	34.3	57%	35.2	48%	34.4	65%	34.6
DYFPEPVTVSWNSGALTSGVHTFPAVLQSSGLYSLSSVVTVPSSSLGTQTYICNVNHKPSNTKVDKKVEPK	K58 or K63	HC-K208, or HC-K213	8535.19			66%	32.8	46%	33.1	44%	32.9	44%	33.1

DYFPEPVTVSWNSGALTSGVHTFPAVLQSSGLYSLSSVVTVPSSSLGTQTYICNVNHKPS NTKVDKKVEPK	K63, K66, or K67	HC-K213, HC-K216, or HC-K217	8535.19	36%	33.0	26%	31.7	51%	31.7	24%	31.5	34%	33.2
EVQLVESGGGLVQPGGSLRLSCAASGFNIKDTYIHWVRQAPGK	K30	HC-K30	5480.70	74%	34.3	74%	34.1	69%	34.2	79%	34.1	71%	34.4
EVQLVESGGGLVQPGGSLRLSCAASGFNIKDTYIHWVRQAPGKGLEWVARIYPTNGYT RYADSVK	K30	HC-K30	8020.98	72%	34.6	72%	34.4	75%	34.5	59%	34.5	64%	34.6
EVQLVESGGGLVQPGGSLRLSCAASGFNIKDTYIHWVRQAPGKGLEWVARIYPTNGYT RYADSVK	K43	HC-K43	8020.98	45%	33.0	58%	32.7	56%	32.9	25%	32.8	52%	33.0
EVQLVESGGGLVQPGGSLRLSCAASGFNIKDTYIHWVRQAPGKGLEWVARIYPTNGYT RYADSVKGRFTISADTSKNTAYLQMNSLRAEDTAVYYCSRWGGDGFYAMDYWGQGT LVTVSSASTKGPSVFPLAPSSK	K30, or K43	HC-K30 or HC-K43	15667.61	25%	35.8	20%	35.7	33%	35.7	36%	35.7	32%	35.8
EVQLVESGGGLVQPGGSLRLSCAASGFNIKDTYIHWVRQAPGKGLEWVARIYPTNGYT RYADSVKGRFTISADTSKNTAYLQMNSLRAEDTAVYYCSRWGGDGFYAMDYWGQGT LVTVSSASTKGPSVFPLAPSSK	K65	HC-K65	15667.61	53%	34.9	43%	34.6	50%	34.9	45%	34.8	50%	34.9
EVQLVESGGGLVQPGGSLRLSCAASGFNIKDTYIHWVRQAPGKGLEWVARIYPTNGYT RYADSVKGRFTISADTSKNTAYLQMNSLRAEDTAVYYCSRWGGDGFYAMDYWGQGT LVTVSSASTKGPSVFPLAPSSK	K76	HC-K76	15667.61	53%	34.9	46%	35.0	47%	34.8	39%	34.8	44%	34.9
EVQLVESGGGLVQPGGSLRLSCAASGFNIKDTYIHWVRQAPGKGLEWVARIYPTNGYT RYADSVKGRFTISADTSKNTAYLQMNSLRAEDTAVYYCSRWGGDGFYAMDYWGQGT LVTVSSASTKGPSVFPLAPSSKSTSGGTAALGCLVK	K65 or K76	HC-K65 or HC-K76	16913.25	11%	35.0	40%	34.6	30%	34.7	24%	34.7	25%	34.7
GLEWVARIYPTNGYTRYADSVKGRFTISADTSKNTAYLQMNSLRAEDTAVYYCSRWG GDGFYAMDYWGQGTLVTVSSASTKGPSVFPLAPSSK	K22	HC-K65	11161.28	42%	36.3	51%	36.1	64%	36.2	68%	36.2	62%	36.3
GLEWVARIYPTNGYTRYADSVKGRFTISADTSKNTAYLQMNSLRAEDTAVYYCSRWG GDGFYAMDYWGQGTLVTVSSASTKGPSVFPLAPSSK	K33	HC-K76	11161.28	48%	36.3	50%	36.1	64%	36.2	71%	36.2	58%	36.2
GQPREPQVYTLPPSREEMTKNQVSLTCLVKGFYPSDIAVEWESNGQPENNYKTTPPVL DSDGSFFLYSKLTVDKSRWQQGNVFSCSVMHEALHNHYTQK	K52	HC-K395	12288.82	58%	32.4			69%	32.4	60%	32.3	59%	32.4
GQPREPQVYTLPPSREEMTKNQVSLTCLVKGFYPSDIAVEWESNGQPENNYKTTPPVL DSDGSFFLYSKLTVDKSRWQQGNVFSCSVMHEALHNHYTQK	K69 or K74	HC-K412 or HC-K417	12288.82	43%	33.4	54%	33.3	45%	33.5	47%	33.2	56%	33.5
GQPREPQVYTLPPSREEMTKNQVSLTCLVKGFYPSDIAVEWESNGQPENNYKTTPPVL DSDGSFFLYSKLTVDKSRWQQGNVFSCSVMHEALHNHYTQKSLSLSPG	K52	HC-K395	12930.16	62%	32.6			49%	32.7	34%	32.8	11%	33.8
GQPREPQVYTLPPSREEMTKNQVSLTCLVKGFYPSDIAVEWESNGQPENNYKTTPPVL DSDGSFFLYSKLTVDKSRWQQGNVFSCSVMHEALHNHYTQKSLSLSPG	K69 or K74	HC-K412 or HC-K417	12930.16	55%	33.7	64%	33.6	39%	33.6	61%	33.5	65%	33.8
GRFTISADTSKNTAYLQMNSLRAEDTAVYYCSRWGGDGFYAMDYWGQGTLVTVSSAS TKGPSVFPLAPSSK	K11	HC-K76	8621.00	73%	37.5	67%	37.3	69%	37.3	76%	37.4	61%	37.2
GRFTISADTSKNTAYLQMNSLRAEDTAVYYCSRWGGDGFYAMDYWGQGTLVTVSSAS TKGPSVFPLAPSSKSTSGGTAALGCLVK	K59	HC-K124	9866.64	46%	36.9			56%	36.9	33%	36.9	30%	37.0
HKVYACEVTHQGLSSPVTK	K2	LC-K190	3039.42	89%	26.3	72%	26.1	89%	26.2	83%	26.0	72%	26.3
HKVYACEVTHQGLSSPVTKSFNRGEC	K19	LC-K207	3832.73	44%	26.7	52%	26.5	52%	26.6	52%	26.4	60%	26.7
LTVDKSRWQQGNVFSCSVMHEALHNHYTQK	K5	HC-K417	4499.06	93%	28.6	93%	28.4	90%	28.5	90%	28.5	86%	28.7
NTAYLQMNSLRAEDTAVYYCSRWGGDGFYAMDYWGQGTLVTVSSASTKGPSVFPLA PSSK	K48	HC-K124	7457.41	37%	38.9	27%	38.8	22%	39.3	46%	38.9	46%	39.0
NTAYLQMNSLRAEDTAVYYCSRWGGDGFYAMDYWGQGTLVTVSSASTKGPSVFPLA PSSKSTSGGTAALGCLVK	K60	HC-K136	8703.05	38%	38.2					38%	38.1	38%	38.3
SCDKTHTCPPCPAPELLGGPSVFLFPPKPK	K4	HC-K225	4118.93	79%	34.1	59%	34.7	66%	34.1	62%	34.0	86%	34.2

STSGGTAALGCLVKDYFPEPVTVSWNSGALTSGVHTFPAVLQSSGLYSLSSVVTVPSSSL GTQTYICNVNHKPSNTKVDKKVEPK	K80, K81, or K85	HC-K216, HC-K217, or HC-K221	9780.83	38%	33.8	30%	33.6	21%	33.7	44%	33.7		
THTCPPCPAPELLGGPSVFLFPPKPK	K24	HC-K249	3685.77	88%	37.9	84%	37.7	72%	37.9	76%	37.7	72%	37.9
THTCPPCPAPELLGGPSVFLFPPKPKDTLMISRTPEVTCVVVDVSHEDPEVK	K26	HC-K251	6565.18	69%	35.4	51%	35.3	57%	35.3	55%	35.3	61%	35.4
THTCPPCPAPELLGGPSVFLFPPKPKDTLMISRTPEVTCVVVDVSHEDPEVKFNWYVDG VEVHNAK	K26	HC-K249	8223.96	43%	35.4	39%	35.2	49%	35.4	29%	35.3	66%	35.4
THTCPPCPAPELLGGPSVFLFPPKPKDTLMISRTPEVTCVVVDVSHEDPEVKFNWYVDG VEVHNAK	K52	HC-K277	8223.96	55%	35.3	49%	35.2	37%	35.3	40%	35.4	54%	35.4
VSNKALPAPIEK	K4	HC-K329	2222.10	82%	30.0	91%	29.8	100 %	29.9	91%	29.8	91%	30.1
TKPREEQYNSTYRVVSVLTVLHQDWLNGK	K2 and G0F N-Glycan	HC-K293 and G0F N-Glycan	5860.69	68%	31.5	61%	32.1	71%	31.4	54%	32.2	71%	31.5
TKPREEQYNSTYRVVSVLTVLHQDWLNGK	K2 and G1F N-Glycan	HC-K293 and G1F N-Glycan	6022.74	68%	31.4	64%	31.1	68%	31.7	82%	31.2	71%	31.4
FNWYVDGVEVHNAKTKPREEQYNSTYRVVSVLTVLHQDWLNGK	K14 or K16 and G0F N-Glycan	HC-K291 or HC-K293 and G0F N-Glycan	7519.47	62%	31.5	76%	31.3	79%	31.4	41%	31.4	64%	31.6
FNWYVDGVEVHNAKTKPREEQYNSTYRVVSVLTVLHQDWLNGK	K14 or K16 and G1F N-Glycan	HC-K291 or HC-K293 and G0F N-Glycan	7681.52	69%	31.5	45%	31.2	74%	31.3	71%	31.3	64%	31.6

Table S2. List of single payload-containing peptides identified with ProSight PD for EThcD replicates in primary sample. For each peptide, the residue to which the payload was localized, the theoretical mass, as well as the sequence coverage and retention time for each replicate are listed. Some replicate entries are blank in the case that a peptide was not identified in all five technical replicates.

Annotated Sequence	Payload Localization (residue number on peptide sequence)	Payload Localization (residue number on protein sequence)	Theo. Mass [Da]	Replicate 1		Replicate 2		Replicate 3		Replicate 4		Replicate 5	
				SC	RT	SC	RT	SC	RT	SC	RT	SC	RT
ADYEKHK	K5	LC-K188	1845.79	100%	25.9	83%	26.1	100%	26.1	100%	26.0	83%	26.1
ADYEKHKVYACEVTHQGLSSPVTK	K5	LC-K188	3645.68	83%	24.9	91%	25.1	83%	25.1	78%	25.0	87%	25.2
ADYEKHKVYACEVTHQGLSSPVTK	K7	LC-K190	3645.68	74%	25.0	57%	25.8	83%	25.8	74%	25.1	61%	25.2
ADYEKHKVYACEVTHQGLSSPVTKSFNRGEC	K5	LC-K188	4439.00	77%	24.8	77%	25.0	80%	25.0	80%	24.9	87%	24.9
ADYEKHKVYACEVTHQGLSSPVTKSFNRGEC	K7	LC-K190	4439.00	57%	25.5	63%	25.7	63%	25.0	67%	25.5	63%	24.9
AKGQPREPQVYTLPPSREEMTK	K2	HC-K343	3497.67	62%	25.9	38%	26.2	57%	26.1	29%	26.1	57%	26.1
AKGQPREPQVYTLPPSREEMTKNQVSLTCLVKGFYPSDIAVEWESNGQPENNYKTTPP VLDSDGSFFLYSKLTVDKSRWQQGNVFSCSVMHEALHNHYTQK	K2	HC-K343	12487.96			14%	30.6	29%	30.4	13%	30.4	12%	30.3
AKGQPREPQVYTLPPSREEMTKNQVSLTCLVKGFYPSDIAVEWESNGQPENNYKTTPP VLDSDGSFFLYSKLTVDKSRWQQGNVFSCSVMHEALHNHYTQK	K54	HC-K395	12487.96	42%	31.5	29%	31.7	50%	31.6	42%	31.5	26%	31.6
AKGQPREPQVYTLPPSREEMTKNQVSLTCLVKGFYPSDIAVEWESNGQPENNYKTTPP VLDSDGSFFLYSKLTVDKSRWQQGNVFSCSVMHEALHNHYTQK	K71 or K76	HC-K412 or HC-K417	12487.96	47%	32.7	45%	32.8	45%	32.7	46%	32.6	55%	32.6
AKGQPREPQVYTLPPSREEMTKNQVSLTCLVKGFYPSDIAVEWESNGQPENNYKTTPP VLDSDGSFFLYSKLTVDKSRWQQGNVFSCSVMHEALHNHYTQKSLSLSPG	K2	HC-K343	13129.29	21%	30.3	26%	30.5	25%	30.5	29%	30.4	8%	30.4
AKGQPREPQVYTLPPSREEMTKNQVSLTCLVKGFYPSDIAVEWESNGQPENNYKTTPP VLDSDGSFFLYSKLTVDKSRWQQGNVFSCSVMHEALHNHYTQKSLSLSPG	K54	HC-K395	13129.29	32%	31.9	31%	32.0	39%	31.8	33%	31.6	44%	31.8
AKGQPREPQVYTLPPSREEMTKNQVSLTCLVKGFYPSDIAVEWESNGQPENNYKTTPP VLDSDGSFFLYSKLTVDKSRWQQGNVFSCSVMHEALHNHYTQKSLSLSPG	K71 or K76	HC-K412 or HC-K417	13129.29	43%	33.1	41%	33.1	41%	33.2	36%	33.3	43%	33.1
ALPAPIEKTISK	K8	HC-K337	2223.12	64%	32.4	55%	32.0					64%	31.8
DIQMTQSPSSLSASVGDRVTITCRASQDVNTAVAWYQQKPGKAPKLLIYSASFLYSGVP SRFSGSRSGTDFTLTISSLQPEDFATYYCQQHYTTPPTFGQGTKVEIKRTVAAPSVFIFPPS DEQLK	K103	LC-K103	14728.24	21%	33.1	18%	33.5	21%	33.3	15%	33.3	9%	33.2
DIQMTQSPSSLSASVGDRVTITCRASQDVNTAVAWYQQKPGKAPKLLIYSASFLYSGVP SRFSGSRSGTDFTLTISSLQPEDFATYYCQQHYTTPPTFGQGTKVEIKRTVAAPSVFIFPPS DEQLK	K39, K42, or K45	LC-K39, LC-K42, or LC-K45	14728.24	28%	34.1	28%	34.1	24%	33.6	23%	34.1	28%	33.9
DSTYSLSSTLTLSKADYEKHKVYACEVTHQGLSSPVTK	K19	LC-K188	5129.42	41%	27.9	65%	28.1	62%	28.1	57%	28.0	57%	28.0
DTLMISRTPEVTCVVVDVSHEDPEVKFNWYVDGVEVHNAK	K26	HC-K277	5512.56	51%	35.6	62%	35.8	49%	35.7	44%	35.6	46%	35.7
DTYIHWVRQAPGKGLEWVARIYPTNGYTRYADSVK	K13	HC-K43	5066.45	24%	31.4	41%	31.6			35%	31.3		
DYFPEPVTVSWNSGALTSGVHTFPAVLQSSGLYSLSSVVTVPSSSLGTQTYICNVNHKPS NTKVDK	K58	HC-K208	7953.84	20%	34.7	34%	34.7	23%	34.6	25%	34.5	17%	34.9
DYFPEPVTVSWNSGALTSGVHTFPAVLQSSGLYSLSSVVTVPSSSLGTQTYICNVNHKPS NTKVDKKVEPK	K58 or K63	HC-K208, or HC-K213	8535.19	46%	33.1	41%	33.2	59%	33.1	31%	33.1	51%	33.1

DYFPEPVTVSWNSGALTSGVHTFPAVLQSSGLYSLSSVVTVPSSSLGTQTYICNVNHKPS NTKVDKKVEPK	K63, K66, or K67	HC-K213, HC-K216, or HC-K217	8535.19	46%	31.6	59%	31.8	30%	31.7	29%	31.6	33%	31.6
EVQLVESGGGLVQPGGSLRLSCAASGFNIKDTYIHWVRQAPGK	K30	HC-K30	5480.70	71%	34.2	76%	34.4	69%	34.5	67%	34.3	74%	34.2
EVQLVESGGGLVQPGGSLRLSCAASGFNIKDTYIHWVRQAPGKGLEWVARIYPTNGYT RYADSVK	K30	HC-K30	8020.98	55%	34.5	59%	34.6	47%	34.7	64%	34.6	59%	34.6
EVQLVESGGGLVQPGGSLRLSCAASGFNIKDTYIHWVRQAPGKGLEWVARIYPTNGYT RYADSVK	K43	HC-K43	8020.98			48%	33.0	53%	33.0	42%	33.0	42%	32.9
EVQLVESGGGLVQPGGSLRLSCAASGFNIKDTYIHWVRQAPGKGLEWVARIYPTNGYT RYADSVKGRFTISADTSKNTAYLQMNSLRAEDTAVYYCSRWGGDGFYAMDYWGQGT LVTVSSASTKGPSVFPLAPSSK	K30	HC-K30	15667.61	19%	35.7	17%	35.9	21%	35.8	22%	35.8	21%	35.8
EVQLVESGGGLVQPGGSLRLSCAASGFNIKDTYIHWVRQAPGKGLEWVARIYPTNGYT RYADSVKGRFTISADTSKNTAYLQMNSLRAEDTAVYYCSRWGGDGFYAMDYWGQGT LVTVSSASTKGPSVFPLAPSSK	K65	HC-K65	15667.61	31%	34.7	30%	35.0	33%	34.8	29%	34.8	30%	34.9
EVQLVESGGGLVQPGGSLRLSCAASGFNIKDTYIHWVRQAPGKGLEWVARIYPTNGYT RYADSVKGRFTISADTSKNTAYLQMNSLRAEDTAVYYCSRWGGDGFYAMDYWGQGT LVTVSSASTKGPSVFPLAPSSK	K76	HC-K76	15667.61	30%	34.7	25%	35.0	33%	34.8	22%	34.8	23%	34.7
EVQLVESGGGLVQPGGSLRLSCAASGFNIKDTYIHWVRQAPGKGLEWVARIYPTNGYT RYADSVKGRFTISADTSKNTAYLQMNSLRAEDTAVYYCSRWGGDGFYAMDYWGQGT LVTVSSASTKGPSVFPLAPSSKSTSGGTAALGCLVK	K65 or K76	HC-K65 or HC-K76	16913.25	9%	34.7	24%	34.8	13%	34.8	26%	34.7		
GLEWVARIYPTNGYTRYADSVKGRFTISADTSKNTAYLQMNSLRAEDTAVYYCSRWG GDGFYAMDYWGQGTLVTVSSASTKGPSVFPLAPSSK	K22	HC-K65	11161.28	34%	36.2	42%	36.3	58%	36.2	55%	36.3	47%	36.2
GLEWVARIYPTNGYTRYADSVKGRFTISADTSKNTAYLQMNSLRAEDTAVYYCSRWG GDGFYAMDYWGQGTLVTVSSASTKGPSVFPLAPSSK	K33	HC-K76	11161.28			37%	36.4			58%	36.3	37%	36.2
GQPREPQVYTLPPSREEMTKNQVSLTCLVKGFYPSDIAVEWESNGQPENNYKTTPPVL DSDGSFFLYSKLTVDKSRWQQGNVFSCSVMHEALHNHYTQK	K52	HC-K395	12288.82	26%	32.6	48%	32.6	36%	32.6	34%	32.3	34%	32.7
GQPREPQVYTLPPSREEMTKNQVSLTCLVKGFYPSDIAVEWESNGQPENNYKTTPPVL DSDGSFFLYSKLTVDKSRWQQGNVFSCSVMHEALHNHYTQK	K69 or K74	HC-K412 or HC-K417	12288.82	39%	33.4	41%	33.5	31%	33.4	30%	33.5	33%	33.5
GQPREPQVYTLPPSREEMTKNQVSLTCLVKGFYPSDIAVEWESNGQPENNYKTTPPVL DSDGSFFLYSKLTVDKSRWQQGNVFSCSVMHEALHNHYTQKSLSLSPG	K69 or K74	HC-K412 or HC-K417	12930.16	33%	33.6	37%	33.9	31%	33.8	22%	33.8	31%	33.6
GRFTISADTSKNTAYLQMNSLRAEDTAVYYCSRWGGDGFYAMDYWGQGTLVTVSSAS TKGPSVFPLAPSSK	K11	HC-K76	8621.00	83%	37.2	86%	37.1	79%	37.3	87%	37.1	81%	37.3
GRFTISADTSKNTAYLQMNSLRAEDTAVYYCSRWGGDGFYAMDYWGQGTLVTVSSAS TKGPSVFPLAPSSKSTSGGTAALGCLVK	K59	HC-K124	9866.64	44%	36.8	52%	37.0			23%	37.3	37%	36.9
HKVYACEVTHQGLSSPVTK	K2	LC-K190	3039.42	78%	26.0	83%	26.3	89%	26.2	78%	26.1	78%	26.1
HKVYACEVTHQGLSSPVTKSFNRGEC	K19	LC-K207	3832.73	40%	26.4	36%	26.7	40%	26.7	56%	26.5		
LTVDKSRWQQGNVFSCSVMHEALHNHYTQK	K5	HC-K417	4499.06	93%	28.4	86%	28.7	90%	28.6	97%	28.5	90%	28.5
NTAYLQMNSLRAEDTAVYYCSRWGGDGFYAMDYWGQGTLVTVSSASTKGPSVFPLA PSSK	K48	HC-K124	7457.41	46%	39.0	61%	39.1	53%	39.4	49%	39.0	58%	38.9
NTAYLQMNSLRAEDTAVYYCSRWGGDGFYAMDYWGQGTLVTVSSASTKGPSVFPLA PSSKSTSGGTAALGCLVK	K60	HC-K136	8703.05	26%	38.1	22%	38.3	11%	38.3	15%	38.5	26%	38.1
SCDKTHTCPPCPAPELLGGPSVFLFPPKPK	K4	HC-K225	4118.93	79%	34.2	83%	34.3	90%	34.2	83%	34.1	90%	34.1
SGTASVVCLLNNFYPREAKVQWKVDNALQSGNSQESVTEQDSKDSTYSLSSTLTLSKA DYEKHKVYACEVTHQGLSSPVTK	K62	LC-K188	9837.70	38%	28.4	43%	28.6	25%	28.5	26%	28.5	40%	28.4
THTCPPCPAPELLGGPSVFLFPPKPK	K24	HC-K249	3685.77	72%	37.7	64%	38.0	64%	38.0	72%	38.0	68%	38.0

THTCPPCPAPELLGGPSVFLFPPKPKDTLMISRTPEVTCVVVDVSHEDPEVK	K24 or K26	HC-K249 or HC-K251	6565.18	43%	35.2	49%	35.4	41%	35.5	37%	35.4	39%	35.4
THTCPPCPAPELLGGPSVFLFPPKPKDTLMISRTPEVTCVVVDVSHEDPEVKFNWYVDGVEVHNAK	K24 or K26	HC-K249 or HC-K251	8223.96	23%	35.5			32%	35.6	26%	35.6	28%	35.5
THTCPPCPAPELLGGPSVFLFPPKPKDTLMISRTPEVTCVVVDVSHEDPEVKFNWYVDGVEVHNAK	K52	HC-K277	8223.96	46%	35.2	43%	35.6	39%	35.4	26%	35.5	45%	35.3
VSNKALPAPIEK	K4	HC-K329	2222.10	91%	29.8	82%	30.1	73%	30.0	91%	29.9	91%	29.9
VYACEVTHQGLSSPVTKSFNRGEC	K17	LC-K207	3567.58	30%	29.8	26%	30.1	26%	30.0	35%	29.9		
TKPREEQYNSTYRVVSVLTVLHQDWLNGK	K2 and G0F N-Glycan	HC-K293 and G0F N-Glycan	5860.69	82%	31.2	82%	31.9	82%	31.8	82%	31.4	64%	31.4
TKPREEQYNSTYRVVSVLTVLHQDWLNGK	K2 and G1F N-Glycan	HC-K293 and G1F N-Glycan	6022.74	71%	31.7	75%	31.4	79%	31.3	75%	31.2	71%	31.3
FNWYVDGVEVHNAKTKPREEQYNSTYRVVSVLTVLHQDWLNGK	K14 and G0F N-Glycan	HC-K291 and G0F N-Glycan	7519.47	71%	31.4	79%	31.6	76%	31.6	64%	31.4	81%	31.4
FNWYVDGVEVHNAKTKPREEQYNSTYRVVSVLTVLHQDWLNGK	K14 and G1F N-Glycan	HC-K291 and G0F N-Glycan	7681.52	64%	31.3	79%	31.5	62%	31.6	71%	31.4	67%	31.4

Table S4. List of two payload-containing peptides identified with ProSight PD for UVPD replicates in primary sample. For each peptide, the residue to which the payload was localized, the theoretical mass, as well as the sequence coverage and retention time for each replicate are listed. Some replicate entries are blank in the case that a peptide was not identified in all five technical replicates.

Annotated Sequence	Payload Localization (residue number on peptide sequence)	Payload Localization (residue number on protein sequence)	Theo. Mass [Da]	Replicate 1		Replicate 2		Replicate 3		Replicate 4		Replicate 5	
				SC	RT	SC	RT	SC	RT	SC	RT	SC	RT
AKGQPREPQVYTLPPSREEMTKNQVSLTCLVKGFYPSDIAVEWESNGQPENNYkTTPPVLDSDGSFFLYSKLTVDkSRWQQGNVFSCSVMHEALHNHYTQK	K54 and K71 or K76	HC-K395 and HC-K412 or HC-K417	13444.32	76%	38.5	78%	38.3	63%	38.3	59%	38.6	40%	38.5
EVQLVESGGGLVQPGGSLRLSCAASGFNIkDTYIHWVRQAPGkGLEWVARIYPTNGYTRYADSVK	K30 and K43	HC-K30 and HC-K43	8977.34	53%	41.8	31%	41.6	16%	41.7	38%	41.7	42%	41.9
GFYPSDIAVEWESNGQPENNYkTTPPVLDSDGSFFLYSKLTVDkSRWQQGNVFSCSVMHEALHNHYTQK	K22 and K44	HC-K395 and HC-K417	9835.44	65%	40.6	40%	40.5	72%	40.6	50%	40.6	69%	40.9
GQPREPQVYTLPPSREEMTKNQVSLTCLVKGFYPSDIAVEWESNGQPENNYkTTPPVLDSDGSFFLYSkLTVDKSRWQQGNVFSCSVMHEALHNHYTQK	K52 and K69 or K74	HC-K395 and HC-K412 or HC-K417	13245.19	17%	39.0					35%	38.8	63%	38.8
NQVSLTCLVKGFYPSDIAVEWESNGQPENNYkTTPPVLDSDGSFFLYSkLTVDKSRWQQGNVFSCSVMHEALHNHYTQK	K32 and K49 or K54	HC-K395 and HC-K412 or HC-K417	10921.03	63%	40.5	35%	40.4	56%	40.5	39%	40.5	51%	40.7
THTCPPCPAPELLGGPSVFLFPPKPkDTLMISRTPEVTCVVVDVSHEDPEVkFNWYVDGVEVHNAK	K26 and K52	HC-K251 and HC-K277	9180.32	25%	41.8	34%	41.8	46%	41.8	28%	41.9	15%	42.0

Table S5. List of two payload-containing peptides identified with ProSight PD for EThcD replicates in primary sample. For each peptide, the residue to which the payload was localized, the theoretical mass, as well as the sequence coverage and retention time for each replicate are listed. Some replicate entries are blank in the case that a peptide was not identified in all five technical replicates.

**Annotated Sequence**	**Payload Localization (residue number on peptide sequence)**	**Payload Localization (residue number on protein sequence)**	**Theo. Mass [Da]**	**Replicate 1**		**Replicate 2**		**Replicate 3**		**Replicate 4**		**Replicate 5**	
				**SC**	**RT**	**SC**	**RT**	**SC**	**RT**	**SC**	**RT**	**SC**	**RT**
ADYEKHKVYACEVTHQGLSSPVTK	K5 and K7	LC-K188 and LC-K190	4602.05	30%	37.3			35%	38.0	35%	37.3		
AKGQPREPQVYTLPPSREEMTKNQVSLTCLVKGFYPSDIAVEWESNGQPENNYkTTPPVLDSDGSFFLYSKLTVDkSRWQQGNVFSCSVMHEALHNHYTQK	K54 and K71 or K76	HC-K395 and HC-K412 or HC-K417	13444.32	38%	38.3	35%	38.0	35%	38.5	38%	38.1	42%	38.3
EVQLVESGGGLVQPGGSLRLSCAASGFNIkDTYIHWVRQAPGkGLEWVARIYPTNGYTRYADSVK	K30 and K43	HC-K30 and HC-K43	8977.34			23%	41.9	20%	41.8	25%	41.9		
GFYPSDIAVEWESNGQPENNYkTTPPVLDSDGSFFLYSKLTVDkSRWQQGNVFSCSVMHEALHNHYTQK	K22 and K44	HC-K395 and HC-K417	9835.44	54%	40.6	56%	40.6	52%	40.6	49%	40.5	56%	40.6
GQPREPQVYTLPPSREEMTKNQVSLTCLVKGFYPSDIAVEWESNGQPENNYkTTPPVLDSDGSFFLYSkLTVDKSRWQQGNVFSCSVMHEALHNHYTQK	K52 and K69 or K74	HC-K395 and HC-K412 or HC-K417	13245.19					28%	38.8	34%	38.9	16%	38.8
NQVSLTCLVKGFYPSDIAVEWESNGQPENNYkTTPPVLDSDGSFFLYSKLTVDkSRWQQGNVFSCSVMHEALHNHYTQK	K32 and K54	HC-K395 and HC-K412 or HC-K417	10921.03	36%	40.5	31%	40.6	45%	40.6	42%	40.5	35%	40.5
THTCPPCPAPELLGGPSVFLFPPkPKDTLMISRTPEVTCVVVDVSHEDPEVkFNWYVDGVEVHNAK	K24 or K26 and K52	HC-K249 or HC-K251 and HC-K277	9180.32	34%	41.9	34%	42.0	29%	42.0	31%	41.8	26%	41.8

Table S6. List of single payload-containing peptides identified with ProSight PD for UVPD replicates in secondary sample. For each peptide, the residue to which the payload was localized, the theoretical mass, as well as the sequence coverage and retention time for each replicate are listed. Some replicate entries are blank in the case that a peptide was not identified in all five technical replicates.

Annotated Sequence	Payload Localization (residue number on peptide sequence)	Payload Localization (residue number on protein sequence)	Theo. Mass [Da]	Replicate 1		Replicate 2		Replicate 3		Replicate 4		Replicate 5	
				SC	RT	SC	RT	SC	RT	SC	RT	SC	RT
ADYEKHK	K5	LC-K188	1845.79	83%	25.6	67%	25.5	83%	25.8	100%	25.8	100%	26.1
ADYEKHKVYACEVTHQGLSSPVTK	K5	LC-K188	3645.68	83%	25.0	70%	24.9	87%	25.1	83%	24.9	87%	25.2
ADYEKHKVYACEVTHQGLSSPVTKSFNRGEC	K5	LC-K188	4439.00	83%	24.7	83%	24.8	80%	24.9	80%	24.8	80%	25.2
AKGQPREPQVYTLPPSREEMTK	K2	HC-K343	3497.66	67%	25.6	67%	25.5	71%	25.7	57%	25.8	76%	26.1
AKGQPREPQVYTLPPSREEMTKNQVSLTCLVKGFYPSDIAVEWESNGQPENNYKTTPPVLDSDGSFFLYSKLTVDKSRWQQGNVFSCSVMHEALHNHYTQK	K32	HC-K373	12487.96	36%	31.4	22%	29.8	24%	31.3	39%	31.4	30%	31.6
AKGQPREPQVYTLPPSREEMTKNQVSLTCLVKGFYPSDIAVEWESNGQPENNYKTTPPVLDSDGSFFLYSKLTVDKSRWQQGNVFSCSVMHEALHNHYTQK	K54	HC-K395	12487.96	39%	31.3	43%	31.1	55%	31.3	48%	31.3	40%	31.6
AKGQPREPQVYTLPPSREEMTKNQVSLTCLVKGFYPSDIAVEWESNGQPENNYKTTPPVLDSDGSFFLYSKLTVDKSRWQQGNVFSCSVMHEALHNHYTQK	K54, K71 or K76	HC-K395, HC-K412, or HC-K417	12487.96	47%	32.5	41%	32.4	34%	32.4	41%	32.5	42%	32.7
AKGQPREPQVYTLPPSREEMTKNQVSLTCLVKGFYPSDIAVEWESNGQPENNYKTTPPVLDSDGSFFLYSKLTVDKSRWQQGNVFSCSVMHEALHNHYTQKSLSLSPG	K71 or K76	HC-K412 or HC-K417	13129.29	35%	32.9	36%	32.7	36%	32.9	35%	33.0	38%	33.1
DIQMTQSPSSLSASVGDRVTITCRASQDVNTAVAWYQQKPGKAPKLLIYSASFLYSGVPSRFSGSRSGTDFTLTISSLQPEDFATYYCQQHYTTPPTFGQGTKVEIKRTVAAPSVFIFPPSDEQLK	K103 or K107	LC-K103 or LC-K107	14728.24	15%	33.0	17%	32.8	17%	32.9			18%	33.2
DIQMTQSPSSLSASVGDRVTITCRASQDVNTAVAWYQQKPGKAPKLLIYSASFLYSGVPSRFSGSRSGTDFTLTISSLQPEDFATYYCQQHYTTPPTFGQGTKVEIKRTVAAPSVFIFPPSDEQLK	K39, K42, or K45	LC-K39, LC-K42, or LC-K45	14728.24	22%	34.1	24%	34.1	19%	33.8	20%	33.8	22%	34.2
DTLMISRTPEVTCVVVDVSHEDPEVKFNWYVDGVEVHNAK	K26	HC-K277	5512.56	74%	35.4	56%	35.3	54%	35.5	59%	35.5	62%	35.5
DYFPEPVTVSWNSGALTSGVHTFPAVLQSSGLYSLSSVVTVPSSSLGTQTYICNVNHKPSNTK	K58	HC-K208	7611.65	21%	36.3	21%	35.7					19%	35.9
EVQLVESGGGLVQPGGSLRLSCAASGFNIKDTYIHWVRQAPGKGLEWVARIYPTNGYTRYADSVKGRFTISADTSKNTAYLQMNSLRAEDTAVYYCSRWGGDGFYAMDYWGQGTLVTVSSASTKGPSVFPLAPSSK	K65 or K76	HC-K65 or HC-K76	15667.61	21%	34.8	14%	34.6			24%	34.8	22%	34.9
EVQLVESGGGLVQPGGSLRLSCAASGFNIKDTYIHWVRQAPGKGLEWVARIYPTNGYTRYADSVKGRFTISADTSKNTAYLQMNSLRAEDTAVYYCSRWGGDGFYAMDYWGQGTLVTVSSASTKGPSVFPLAPSSKSTSGGTAALGCLVK	K65 or K76	HC-K65 or HC-K76	16913.25	22%	34.7			19%	34.7	24%	34.7	19%	34.8
EVQLVESGGGLVQPGGSLRLSCAASGFNIKDTYIHWVRQAPGKGLEWVARIYPTNGYTRYADSVK	K30 or K43	HC-K30 or HC-K43	8020.98	16%	32.7	20%	34.4	25%	34.5				
EVQLVESGGGLVQPGGSLRLSCAASGFNIKDTYIHWVRQAPGK	K30	HC-K30	5480.70	60%	34.0	52%	34.1	52%	34.3	50%	34.1	55%	34.2
GFYPSDIAVEWESNGQPENNYKTTPPVLDSDGSFFLYSKLTVDKSRWQQGNVFSCSVMHEALHNHYTQK	K39 or K44	HC-K412 or HC-K417	8879.08	43%	34.2			27%	34.3	47%	34.2	27%	34.4

GLEWVARIYPTNGYTRYADSVKGRFTISADTSKNTAYLQMNSLRAEDTAVYYCSRWGGDGFYAMDYWGQGTLVTVSSASTKGPSVFPLAPSSK	K22	HC-K65	11161.28	37%	36.2	42%	36.0	24%	36.2	37%	36.1	40%	36.2
GLEWVARIYPTNGYTRYADSVKGRFTISADTSKNTAYLQMNSLRAEDTAVYYCSRWGGDGFYAMDYWGQGTLVTVSSASTKGPSVFPLAPSSK	K33	HC-K76	11161.28	37%	36.2	42%	36.0	35%	36.2	36%	36.1	39%	36.2
GQPREPQVYTLPPSREEMTKNQVSLTCLVKGFYPSDIAVEWESNGQPENNYKTTPPVLDSDGSFFLYSKLTVDKSRWQQGNVFSCSVMHEALHNHYTQK	K52	HC-395	12288.82	40%	32.3	29%	32.9	35%	32.2	34%	32.2	35%	32.4
GQPREPQVYTLPPSREEMTKNQVSLTCLVKGFYPSDIAVEWESNGQPENNYKTTPPVLDSDGSFFLYSKLTVDKSRWQQGNVFSCSVMHEALHNHYTQK	K69 or K74	HC-K412 or HC-K417	12288.82	36%	33.1	29%	32.9	31%	33.2	22%	33.3	31%	33.3
GRFTISADTSKNTAYLQMNSLRAEDTAVYYCSRWGGDGFYAMDYWGQGTLVTVSSASTKGPSVFPLAPSSK	K11	HC-K76	8621.00	56%	37.3	54%	37.0	50%	37.3	53%	37.4	56%	37.3
GRFTISADTSKNTAYLQMNSLRAEDTAVYYCSRWGGDGFYAMDYWGQGTLVTVSSASTKGPSVFPLAPSSKSTSGGTAALGCLVK	K59 or K71	HC-K124 or HC-K136	9866.64	14%	36.8	38%	36.8	14%	36.8			18%	36.9
HKVYACEVTHQGLSSPVTK	K2	LC-K190	3039.42	67%	25.6	56%	25.7	67%	25.9	78%	25.9	61%	26.2
LTVDKSRWQQGNVFSCSVMHEALHNHYTQK	K5	HC-K417	4499.06	93%	28.2	97%	27.8	97%	28.2	93%	28.2	86%	28.8
NTAYLQMNSLRAEDTAVYYCSRWGGDGFYAMDYWGQGTLVTVSSASTKGPSVFPLAPSSK	K48	HC-K124	7457.41	32%	39.3	39%	38.8	44%	38.9	34%	39.0	42%	38.9
NTAYLQMNSLRAEDTAVYYCSRWGGDGFYAMDYWGQGTLVTVSSASTKGPSVFPLAPSSKSTSGGTAALGCLVK	K60	HC-K136	8703.05	27%	38.2	27%	38.1	36%	38.1	36%	38.1	38%	38.1
SCDKTHTCPPCPAPELLGGPSVFLFPPKPK	K4	HC-K225	4118.93	69%	34.5	69%	34.0	69%	34.1	66%	33.9	66%	34.0
SGTASVVCLLNNFYPREAKVQWKVDNALQSGNSQESVTEQDSKDSTYSLSSTLTLSKADYEKHKVYACEVTHQGLSSPVTKSFNRGEC	K57, K62, or K64	LC-K183, LC-K188, or LC-K19	10631.02	26%	27.7	28%	27.6	40%	27.9	32%	28.1	30%	28.3
THTCPPCPAPELLGGPSVFLFPPKPKDTLMISRTPEVTCVVVDVSHEDPEVKFNWYVDGVEVHNAK	K24, or K26	HC-K249 or HC-K251	8223.96	34%	35.3	35%	35.1	29%	35.1	28%	35.1	25%	35.4
THTCPPCPAPELLGGPSVFLFPPKPKDTLMISRTPEVTCVVVDVSHEDPEVKFNWYVDGVEVHNAK	K52	HC-K277	8223.96	26%	35.3	26%	35.0	17%	35.2	45%	35.0	37%	35.2
THTCPPCPAPELLGGPSVFLFPPKPKDTLMISRTPEVTCVVVDVSHEDPEVK	K26	HC-K251	6565.18	35%	35.4	45%	35.0	41%	35.1	53%	35.1	45%	35.2
THTCPPCPAPELLGGPSVFLFPPKPK	K24	HC-K249	3685.77	72%	37.7	60%	37.7	72%	37.7	60%	37.9	72%	37.8
VSNKALPAPIEK	K4	HC-K329	2222.10	82%	29.4	82%	29.3	82%	29.6	64%	29.6	73%	30.0
VYACEVTHQGLSSPVTKSFNRGEC	K17	LC-K207	3567.58	44%	29.5	48%	29.4	39%	29.6	35%	29.7	39%	30.0
TKPREEQYNSTYRVVSVLTVLHQDWLNGK	K2 and G0F N-Glycan	HC-K293 and G0F N-Glycan	5860.69	54%	31.8	61%	30.6	54%	31.1	54%	31.8	50%	31.9
TKPREEQYNSTYRVVSVLTVLHQDWLNGK	K2 and G1F N-Glycan	HC-K293 and G1F N-Glycan	6022.74	50%	30.9	64%	30.7	57%	31.0	54%	31.0	54%	31.9
DTLMISRTPEVTCVVVDVSHEDPEVKFNWYVDGVEVHNAKTKPREEQYNSTYRVVSVLTVLHQDWLNGK	K40 or K42 and G0F N-Glycan	HC-K291 or HC-K293 and G0F N-Glycan	10398.88			28%	31.8	31%	31.6	34%	31.6	32%	31.7
FNWYVDGVEVHNAKTKPREEQYNSTYRVVSVLTVLHQDWLNGK	K14 or K16 and G0F N-Glycan	HC-K291 or HC-K293 and G0F N-Glycan	7519.47	67%	31.1			45%	31.2	52%	31.2	48%	31.4
FNWYVDGVEVHNAKTKPREEQYNSTYRVVSVLTVLHQDWLNGK	K14 or K16 and G1F N-Glycan	HC-K291 or HC-K293 and G1F N-Glycan	7681.52	38%	31.0	41%	30.8	31%	31.1	50%	31.2	41%	31.3

Table S7. List of single payload-containing peptides identified with ProSight PD for EThcD replicates in secondary sample. For each peptide, the residue to which the payload was localized, the theoretical mass, as well as the sequence coverage and retention time for each replicate are listed. Some replicate entries are blank in the case that a peptide was not identified in all five technical replicates.

Annotated Sequence	Payload Localization (residue number on peptide sequence)	Payload Localization (residue number on protein sequence)	Theo. Mass [Da]	Replicate 1		Replicate 2		Replicate 3		Replicate 4		Replicate 5	
				SC	RT	SC	RT	SC	RT	SC	RT	SC	RT
ADYEKHK	K5	LC-K188	1845.79	100%	25.9	83%	26.0	100%	26.6	100%	26.7	100%	26.0
ADYEKHKVYACEVTHQGLSSPVTK	K5	LC-K188	3645.68	74%	24.9	87%	25.1	83%	25.1	96%	25.2	87%	25.1
ADYEKHKVYACEVTHQGLSSPVTK	K7	LC-K190	3645.68	30%	25.0	35%	25.8			83%	25.7	74%	25.8
ADYEKHKVYACEVTHQGLSSPVTKSFNRGEC	K5	LC-K188	4439.00	80%	24.7	80%	25.1	77%	24.8	77%	25.0	80%	24.9
ADYEKHKVYACEVTHQGLSSPVTKSFNRGEC	K7	LC-K190	4439.00	57%	24.7	57%	25.6	73%	25.7	63%	25.6	57%	24.9
AKGQPREPQVYTLPPSREEMTK	K2	HC-K343	3497.66	67%	25.8	48%	26.4	62%	26.1	67%	26.4	67%	26.2
AKGQPREPQVYTLPPSREEMTKNQVSLTCLVKGFYPSDIAVEWESNGQPENNYKTTPPVLDSDGSFFLYSKLTVDKSRWQQGNVFSCSVMHEALHNHYTQK	K2	HC-K343	12487.96	25%	30.2	8%	30.4			17%	30.2	18%	30.4
AKGQPREPQVYTLPPSREEMTKNQVSLTCLVKGFYPSDIAVEWESNGQPENNYKTTPPVLDSDGSFFLYSKLTVDKSRWQQGNVFSCSVMHEALHNHYTQK	K54	HC-K395	12487.96	46%	31.4	40%	31.6	40%	31.5	39%	31.7	43%	31.6
AKGQPREPQVYTLPPSREEMTKNQVSLTCLVKGFYPSDIAVEWESNGQPENNYKTTPPVLDSDGSFFLYSKLTVDKSRWQQGNVFSCSVMHEALHNHYTQK	K71 or K76	HC-K412 or HC-K417	12487.96	46%	32.5	47%	32.7	50%	32.6	47%	32.8	50%	32.6
AKGQPREPQVYTLPPSREEMTKNQVSLTCLVKGFYPSDIAVEWESNGQPENNYKTTPPVLDSDGSFFLYSKLTVDKSRWQQGNVFSCSVMHEALHNHYTQKSLSLSPG	K54	HC-K395	13129.29	37%	31.6	38%	31.7			47%	31.7	33%	32.1
AKGQPREPQVYTLPPSREEMTKNQVSLTCLVKGFYPSDIAVEWESNGQPENNYKTTPPVLDSDGSFFLYSKLTVDKSRWQQGNVFSCSVMHEALHNHYTQKSLSLSPG	K71 or K76	HC-K412 or HC-K417	13129.29	40%	33.0	44%	33.1	36%	33.1	41%	33.1	38%	33.2
ALPAPIEKTISK	K8	HC-K337	2223.12	73%	31.6	73%	31.8	55%	31.6	73%	31.8	73%	32.5
DIQMTQSPSSLSASVGDRVTITCRASQDVNTAVAWYQQKPGKAPKLLIYSASFLYSGVPSRFSGSRSGTDFTLTISSLQPEDFATYYCQQHYTTPPTFGQGTKVEIKRTVAAPSVFIFPPSDEQLK	K38, K42, or K45	LC-K39, LC-K42, or LC-K45	14728.24	26%	33.8	22%	33.8	29%	33.8	26%	34.0	21%	34.1
DIQMTQSPSSLSASVGDRVTITCRASQDVNTAVAWYQQKPGKAPKLLIYSASFLYSGVPSRFSGSRSGTDFTLTISSLQPEDFATYYCQQHYTTPPTFGQGTKVEIKRTVAAPSVFIFPPSDEQLK	K103 or K107	LC-K103 or LC-K107	14728.24	16%	33.1	16%	33.0	20%	33.0	18%	33.3	15%	33.1
DSTYSLSSTLTLSKADYEKHKVYACEVTHQGLSSPVTKSFNRGEC	K19 or K21	LC-K188 or LC-K190	5922.74	43%	27.9	18%	28.1	66%	27.7	30%	28.2	34%	28.2
DTLMISRTPEVTCVVVDVSHEDPEVKFNWYVDGVEVHNAK	K26	HC-K277	5512.56	54%	35.5	54%	35.5	39%	35.4	54%	35.6	62%	35.5
DTYIHWVRQAPGKGLEWVARIYPTNGYTRYADSVK	K13	HC-K43	5066.45			62%	31.3	56%	31.3	59%	31.4		
DYFPEPVTVSWNSGALTSGVHTFPAVLQSSGLYSLSSVVTVPSSSLGTQTYICNVNHKPSNTK	K58	HC-K208	7611.65	37%	35.8	29%	35.9	18%	35.9	23%	36.0		
EVQLVESGGGLVQPGGSLRLSCAASGFNIKDTYIHWVRQAPGK	K30	HC-K30	5480.70	67%	34.1	64%	34.2	67%	34.2	62%	34.3	55%	34.2
EVQLVESGGGLVQPGGSLRLSCAASGFNIKDTYIHWVRQAPGKGLEWVARIYPTNGYTRYADSVK	K30	HC-K30	8020.98	31%	34.4	23%	34.5	44%	34.5	50%	34.6	45%	34.5

EVQLVESGGGLVQPGGSLRLSCAASGFNIKDTYIHWVRQAPGKGLEWVARIYPTNGYTRYADSVKGRFTISADTSKNTAYLQMNSLRAEDTAVYYCSRWGGDGFYAMDYWGQGTLVTVSSASTKGPSVFPLAPSSK	K65 or K76	HC-K65 or HC-K76	15667.61	22%	34.8	23%	34.8	13%	34.9	16%	35.0	23%	34.9
GFYPSDIAVEWESNGQPENNYKTTPPVLDSDGSFFLYSKLTVDKSRWQQGNVFSCSVMHEALHNHYTQK	K44	HC-K417	8879.08	59%	34.2	52%	34.3	44%	34.3	44%	34.4	46%	34.3
GLEWVARIYPTNGYTRYADSVKGRFTISADTSKNTAYLQMNSLRAEDTAVYYCSRWGGDGFYAMDYWGQGTLVTVSSASTKGPSVFPLAPSSK	K22	HC-K65	11161.28	26%	36.1	47%	36.1	55%	36.2	45%	36.3	44%	36.2
GLEWVARIYPTNGYTRYADSVKGRFTISADTSKNTAYLQMNSLRAEDTAVYYCSRWGGDGFYAMDYWGQGTLVTVSSASTKGPSVFPLAPSSK	K33	HC-K76	11161.28	26%	36.1	47%	36.1	41%	36.1			44%	36.2
GQPREPQVYTLPPSREEMTKNQVSLTCLVKGFYPSDIAVEWESNGQPENNYKTTPPVLDSDGSFFLYSKLTVDKSRWQQGNVFSCSVMHEALHNHYTQK	K52	HC-395	12288.82	36%	32.2	42%	32.5	32%	32.4	38%	32.6	34%	32.4
GQPREPQVYTLPPSREEMTKNQVSLTCLVKGFYPSDIAVEWESNGQPENNYKTTPPVLDSDGSFFLYSKLTVDKSRWQQGNVFSCSVMHEALHNHYTQK	K69 or K74	HC-K412 or HC-K417	12288.82	35%	33.2	21%	33.4	36%	33.2	33%	33.5	17%	33.6
GRFTISADTSKNTAYLQMNSLRAEDTAVYYCSRWGGDGFYAMDYWGQGTLVTVSSASTKGPSVFPLAPSSK	K11	HC-K76	8621.00	87%	37.3	80%	37.3	74%	37.3	69%	37.7	87%	37.2
HKVYACEVTHQGLSSPVTK	K2	LC-K190	3039.42	83%	25.9	78%	26.2	83%	26.2	83%	26.2	89%	26.2
LTVDKSRWQQGNVFSCSVMHEALHNHYTQK	K5	HC-K417	4499.06	90%	28.3	93%	28.7	90%	28.7	90%	28.6	97%	28.6
NTAYLQMNSLRAEDTAVYYCSRWGGDGFYAMDYWGQGTLVTVSSASTKGPSVFPLAPSSK	K48	HC-K124	7457.41	63%	38.9	44%	38.9	49%	38.8	56%	38.9	51%	38.8
NTAYLQMNSLRAEDTAVYYCSRWGGDGFYAMDYWGQGTLVTVSSASTKGPSVFPLAPSSKSTSGGTAALGCLVK	K60	HC-K136	8703.05	22%	38.1	23%	38.1	21%	38.0	19%	38.2	19%	38.1
SCDKTHTCPPCPAPELLGGPSVFLFPPKPK	K4	HC-K225	4118.93	86%	33.9	83%	33.9	83%	34.0	76%	34.0	76%	34.1
SGTASVVCLLNNFYPREAKVQWKVDNALQSGNSQESVTEQDSKDSTYSLSSTLTLSKADYEKHKVYACEVTHQGLSSPVTKSFNRGEC	K62	LC-K188	10631.02	31%	28.1	22%	28.4	26%	28.4	22%	28.4	36%	28.4
THTCPPCPAPELLGGPSVFLFPPKPK	K24	HC-K249	3685.77	68%	37.7	88%	37.8	68%	37.6	68%	37.8	68%	37.7
THTCPPCPAPELLGGPSVFLFPPKPKDTLMISRTPEVTCVVVDVSHEDPEVK	K24 or K26	HC-K249 or HC-K251	6565.18	53%	35.1	31%	35.5	49%	35.1	37%	35.3	33%	35.2
THTCPPCPAPELLGGPSVFLFPPKPKDTLMISRTPEVTCVVVDVSHEDPEVKFNWYVDGVEVHNAK	K26	HC-K251	8223.96	31%	35.2	31%	35.4	15%	35.4	22%	35.4	31%	35.4
THTCPPCPAPELLGGPSVFLFPPKPKDTLMISRTPEVTCVVVDVSHEDPEVKFNWYVDGVEVHNAK	K52	HC-K277	8223.96	45%	35.1	32%	35.3	40%	35.2	40%	35.3	32%	35.4
VSNKALPAPIEK	K4	HC-K329	2222.10	91%	29.8	100%	29.9	73%	29.9	91%	30.0	82%	29.9
VYACEVTHQGLSSPVTKSFNRGEC	K17	LC-K207	3567.58					35%	30.0	35%	30.0	26%	30.1
TKPREEQYNSTYRVVSVLTVLHQDWLNGK	K2 and G0F N-Glycan	HC-K293 and G0F N-Glycan	5860.69	79%	31.2	68%	31.9	75%	31.4	79%	31.4	61%	31.4
TKPREEQYNSTYRVVSVLTVLHQDWLNGK	K2 and G1F N-Glycan	HC-K293 and G1F N-Glycan	6022.74	86%	31.1	79%	31.3	75%	31.3	82%	31.4	71%	31.3
DTLMISRTPEVTCVVVDVSHEDPEVKFNWYVDGVEVHNAKTKPREEQYNSTYRVVSVLTVLHQDWLNGK	K40 and G0F N-Glycan	HC-K291 and G0F N-Glycan	10398.88	29%	31.6	47%	31.8	52%	31.7	41%	31.8	22%	31.7
FNWYVDGVEVHNAKTKPREEQYNSTYRVVSVLTVLHQDWLNGK	K14 and G0F N-Glycan	HC-K291 and G0F N-Glycan	7519.47	86%	31.3	64%	31.5	74%	31.4	79%	31.5	55%	31.5
FNWYVDGVEVHNAKTKPREEQYNSTYRVVSVLTVLHQDWLNGK	K14 and G1F N-Glycan	HC-K291 and G1F N-Glycan	7681.52	71%	31.3	71%	31.4	81%	31.3	81%	31.4	48%	31.4

Table S8. List of two payload-containing peptides identified with ProSight PD for UVPD replicates in secondary sample. For each peptide, the residue to which the payload was localized, the theoretical mass, as well as the sequence coverage and retention time for each replicate are listed. Some replicate entries are blank in the case that a peptide was not identified in all five technical replicates.

Annotated Sequence	Payload Localization (residue number on peptide sequence)	Payload Localization (residue number on protein sequence)	Theo. Mass [Da]	Replicate 1		Replicate 2		Replicate 3		Replicate 4		Replicate 5	
				SC	RT	SC	RT	SC	RT	SC	RT	SC	RT
AKGQPREPQVYTLPPSREEMTKNQVSLTCLVKGFYPSDIAVEWESNGQPENNYkTTPPVLDSDGSFFLYSkLTVDKSRWQQGNVFSCSVMHEALHNHYTQK	K54 and K71 or K76	HC-K395 and HC-K412 or HC-K417	13444.32	29%	38.5	35%	38.3	34%	38.3	45%	38.2	36%	38.3
GFYPSDIAVEWESNGQPENNYkTTPPVLDSDGSFFLYSkLTVDKSRWQQGNVFSCSVMHEALHNHYTQK	K22 and K39 or K44	HC-K395 and HC-K412 or HC-K417	9835.44	37%	40.6	41%	40.5	34%	40.6	18%	40.5	21%	40.5
GQPREPQVYTLPPSREEMTKNQVSLTCLVKGFYPSDIAVEWESNGQPENNYkTTPPVLDSDGSFFLYSkLTVDKSRWQQGNVFSCSVMHEALHNHYTQK	K52 and K69 or K74	HC-K395 and HC-K412 or HC-K417	13245.19	19%	38.9			25%	38.8			30%	38.8
NQVSLTCLVKGFYPSDIAVEWESNGQPENNYkTTPPVLDSDGSFFLYSkLTVDKSRWQQGNVFSCSVMHEALHNHYTQK	K32 and K49 or K54	HC-K395 and HC-K412 or HC-K417	10921.03	30%	40.6	23%	40.7	31%	40.7	40%	40.5	23%	40.5
THTCPPCPAPELLGGPSVFLFPPkPKDTLMISRTPEVTCVVVDVSHEDPEVkFNWYVDGVEVHNAK	K24 or K26 and K52	HC-K249 or HC-K251 and HC-K277	9180.32			22%	42.0	#N/A	#N/A	26%	42.0	25%	42.1

Table S9. List of two payload-containing peptides identified with ProSight PD for EThcD replicates in secondary sample. For each peptide, the residue to which the payload was localized, the theoretical mass, as well as the sequence coverage and retention time for each replicate are listed. Some replicate entries are blank in the case that a peptide was not identified in all five technical replicates.

Annotated Sequence	Payload Localization (residue number on peptide sequence)	Payload Localization (residue number on protein sequence)	Theo. Mass [Da]	Replicate 1		Replicate 2		Replicate 3		Replicate 4		Replicate 5	
				SC	RT	SC	RT	SC	RT	SC	RT	SC	RT
AKGQPREPQVYTLPPSREEMTKNQVSLTCLVKGFYPSDIAVEWESNGQPENNYkTTPPVLDSDGSFFLYSkLTVDKSRWQQGNVFSCSVMHEALHNHYTQK	K54 and K71 or K76	HC-K395 and HC-K412 or HC-K417	13444.32	33%	38.4	40%	38.0	37%	38.2	38%	38.4	38%	38.3
EVQLVESGGGLVQPGGSLRLSCAASGFNIkDTYIHWVRQAPGkGLEWVARIYPTNGYTRYADSVK	K30 and K43	HC-K30 and HC-K43	8977.34	23%	41.8	33%	41.7	17%	41.7	25%	41.9	28%	41.8
GFYPSDIAVEWESNGQPENNYkTTPPVLDSDGSFFLYSKLTVDkSRWQQGNVFSCSVMHEALHNHYTQK	K22 and K44	HC-K395 and HC-K417	9835.44	46%	40.5	63%	40.8	46%	40.5	49%	40.7	57%	40.5
NQVSLTCLVKGFYPSDIAVEWESNGQPENNYkTTPPVLDSDGSFFLYSKLTVDkSRWQQGNVFSCSVMHEALHNHYTQK	K32 and K49 or K54	HC-K395 and HC-K412 or HC-K417	10921.03	33%	40.5	39%	40.5	35%	40.4	32%	40.5	40%	40.4
THTCPPCPAPELLGGPSVFLFPPkPKDTLMISRTPEVTCVVVDVSHEDPEVkFNWYVDGVEVHNAK	K24 or K26 and K52	HC-K249 or HC-K251 and HC-K277	9180.32	20%	42.0	43%	41.8	32%	41.8	39%	41.8	31%	41.8

Table S10. Number of identified payload-modified peptide spectral matches for two different lots of TDM-1 based on bottom-up analysis of tryptic digests.

Number of payload-modified peptide spectral matches		
	**Lot #3535802**	**Lot #1153402**
**HCD only**	21	46
**HCD-triggered-EThcD**	69*	48*
**HCD-triggered-UVPD**	75*	76*
No score cut-off is used<br>* Includes peptide spectral matches (PSMs) from HCD as well.		

Table S11. Number of payload-modified lysines identified in heavy and light chains (HC and LC) of two different lots of TDM-1 based on bottom-up analysis of tryptic digests.

**Number of modified Lysines (HC + LC)**		
	**Lot #3535802**	**Lot #1153402**
**HCD only**	7 + 0	11 + 5
**HCD-triggered-EThcD**	12 + 5 *	13 + 3 *
**HCD-triggered-UVPD**	14 + 4 *	15 + 5*

No score cut-off is used
* Includes peptide spectral matches (PSMs) from HCD as well.
